# Organellar genome assembly methods and comparative analysis of horticultural plants

**DOI:** 10.1038/s41438-017-0002-1

**Published:** 2018-01-10

**Authors:** Xuelin Wang, Feng Cheng, Dekai Rohlsen, Changwei Bi, Chunyan Wang, Yiqing Xu, Suyun Wei, Qiaolin Ye, Tongming Yin, Ning Ye

**Affiliations:** 1 College of Information Science and Technology, Nanjing Forestry University, Nanjing, Jiangsu China; 2 Department of Pharmaceutical Science, College of Pharmacy, University of South Florida, Tampa, FL 33612 USA; 3 School of Biological Science and Medical Engineering, Southeast University, Nanjing, Jiangsu China; 4 College of Forestry, Nanjing Forestry University, Nanjing, Jiangsu China

## Abstract

Although organellar genomes (including chloroplast and mitochondrial genomes) are smaller than nuclear genomes in size and gene number, organellar genomes are very important for the investigation of plant evolution and molecular ecology mechanisms. Few studies have focused on the organellar genomes of horticultural plants. Approximately 1193 chloroplast genomes and 199 mitochondrial genomes of land plants are available in the National Center for Biotechnology Information (NCBI), of which only 39 are from horticultural plants. In this paper, we report an innovative and efficient method for high-quality horticultural organellar genome assembly from next-generation sequencing (NGS) data. Sequencing reads were first assembled by Newbler, Amos, and Minimus software with default parameters. The remaining gaps were then filled through BLASTN search and PCR. The complete DNA sequence was corrected based on Illumina sequencing data using BWA (Burrows–Wheeler Alignment tool) software. The advantage of this approach is that there is no need to isolate organellar DNA from total DNA during sample preparation. Using this procedure, the complete mitochondrial and chloroplast genomes of an ornamental plant, *Salix suchowensis*, and a fruit tree, *Ziziphus jujuba*, were identified. This study shows that horticultural plants have similar mitochondrial and chloroplast sequence organization to other seed plants. Most horticultural plants demonstrate a slight bias toward A+T rich features in the mitochondrial genome. In addition, a phylogenetic analysis of 39 horticultural plants based on 15 protein-coding genes showed that some mitochondrial genes are horizontally transferred from chloroplast DNA. Our study will provide an important reference for organellar genome assembly in other horticultural plants. Furthermore, phylogenetic analysis of the organellar genomes of horticultural plants could accurately clarify the unanticipated relationships among these plants.

## Genetics: Innovative DNA assembly method for mitochondrial and chloroplast genomes

A new method makes it easier to assemble the DNA sequences of mitochondrial and chloroplast genomes for horticultural plants. Most strategies for assembling the genomes of chloroplasts (which conduct photosynthesis) and mitochondria (which supply cellular energy) require the isolation of organellar DNA from total DNA. However, using bioinformatics software, a team led by Tongming Yin and Ning Ye from Nanjing Forestry University, China, identified a way to pull out all the DNA sequence reads for these organelles from complete next-generation sequencing data, without the need for specialized sample preparation. As a proof-of-principle, the researchers used the protocol to decode the mitochondrial and chloroplast genomes of the ornamental plant *Salix suchowensis* and the fruit tree *Ziziphus jujube*. Comparative analyses of mitochondrial and chloroplast genomes assembled in this way could reveal new insights into plant evolutionary relationships.

## Introduction

Horticultural plants, which are grown for aesthetic value or as food in a home garden, can improve mental and physical health^1^. In plant cells, chloroplasts and mitochondria are the necessary organelles forming the powerhouse of the cell. Chloroplasts conduct photosynthesis, and mitochondria indirectly supply energy. In addition, both possess their own DNA. A horticultural plant cell generally has one copy of the nuclear genome and multiple copies of organellar genomes (including chloroplast and mitochondrial genomes). For example, the plastid genome in plant leaf cells has 400 to 1600 copies^2^. The chloroplast genomes of horticultural plants are highly conserved and possess a circular DNA structure varying from 120^3^ to 163 kb^4^. The chloroplast genomes of horticultural species consist of four parts, two copies of inverted repeats (IR) of 20–28 kb in size, an LSC (large single-copy) area of 80–90 kb, and an SSC (small single-copy) area of 16–27 kb^5^. The LSC and SSC areas are separated by the IRs. The mitochondrial genomes of horticultural plants are very complex and have distinct characteristics including large genome size, foreign DNA uptake, and continued recombination^6^. As a result of non-coding sequence extension and a large repetitive section^7^, the lengths of the published mitochondrial genomes of angiosperms, especially horticultural plants, vary in size^8,9^, ranging from 258 kb in *Raphanus sativus*^10^ to 983 kb in *Cucurbita pepo*^11^. Sequence data of plant organellar genomes are accumulating at a very rapid pace. Currently, over 1193 chloroplast and 199 mitochondrial genome sequences of land plants are included in the NCBI GenBank Organelle Genome Resources (http://www.ncbi.nlm.nih.gov/genome/browse/). However, only 39 organellar genomes of horticultural plants are present in the database.

Most strategies for assembling organellar genomes require the isolation of chloroplast or mitochondrial DNA from total DNA during the sample preparation. For chloroplast genome assembly, one of the time-consuming steps in the traditional method is to extend overlapping fragments by the polymerase chain reaction (PCR) from conserved gene loci. An alternative approach is to first isolate chloroplasts and then identify sequences using high-throughput sequencing techniques^12^. Similarly, there are several approaches for mitochondrial genome assembly. For example, Unseld *et al*. determined the sequence of the mitochondrial DNA of *Arabidopsis thaliana* using a shotgun-based approach. Mitochondrial DNA was first isolated from cosmid libraries of total *Arabidopsis thaliana* DNA. Random fragments were obtained from entire trimmed and subcloned cosmids. These fragments were then sequenced and assembled into contigs for unique mitochondrial sequences^13^. There are other two strategies for mitochondrial genome assembly: physical map-based^14^ and gene-based^15^. For these methods, the key step is isolating organellar DNA. However, this step is challenging and time consuming^16^. In addition, the large size of replication and the dynamic nature of the mitochondrial genome, including foreign DNA uptake and genome recombination, make the sequence assembly complex.

Next-generation sequencing (NGS) technologies using Roche or Illumina platforms provide new high-throughput, low-cost, and efficient methods for chloroplast and mitochondrial genome assembly^17–19^. In this paper, we introduce an innovative and efficient method for de novo horticultural organellar genome assembly from next generation whole-genome sequencing data without organellar DNA isolation. We have successfully assembled the complete chloroplast and mitochondrial genomes of an ornamental plant, *Salix suchowensis*, and a fruit tree, *Ziziphus jujube*, which is the first plant in the Rhamnaceae family to have its chloroplast genome sequenced^20^. Whole-genome sequencing of these two plants was conducted at Nanjing Forestry University. Our study paves the way for the organellar genome assemblies of other horticultural plants^21^.

## Materials and methods

**Fig. 1:**
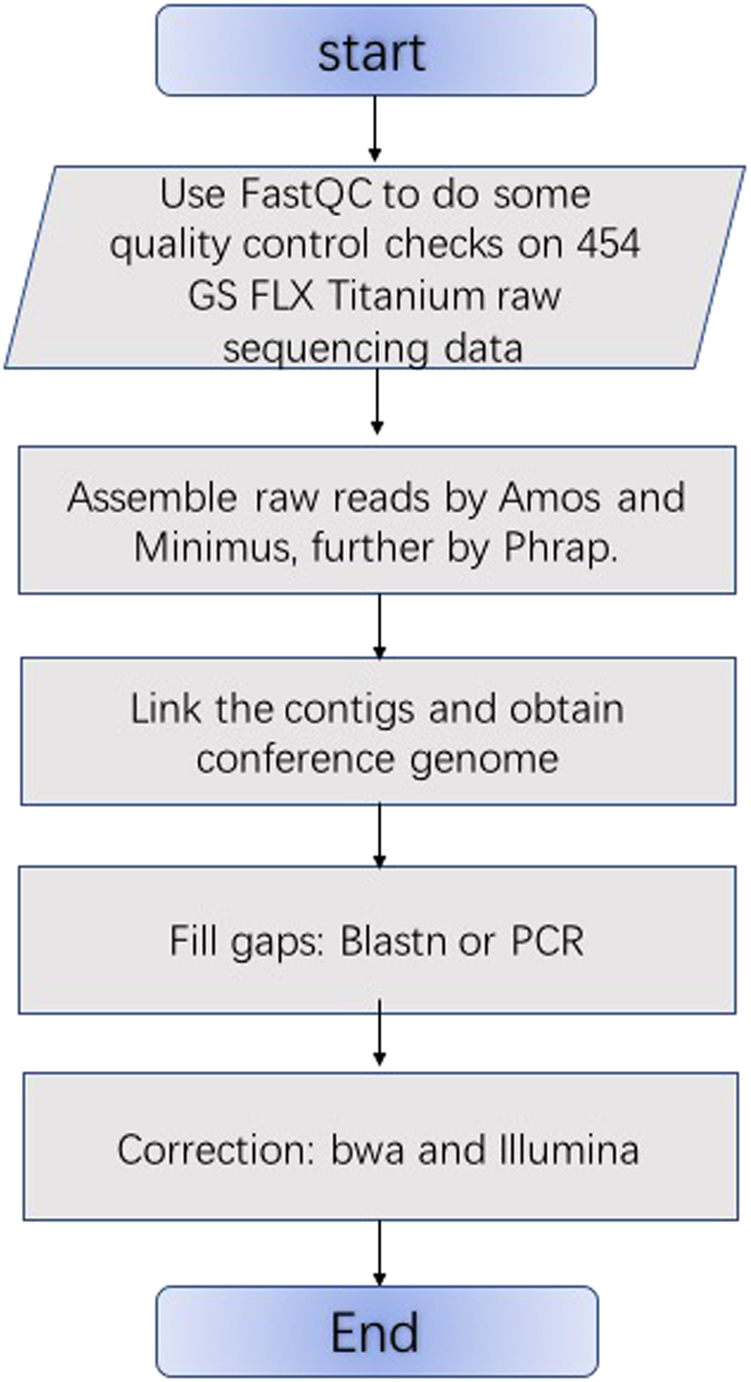
The pipeline flow chart of the assembly of the chloroplast genome of *Ziziphus jujuba*. The assembly process includes data preparation, assembly of raw reads, the creation of the contig graph, and the construction of the chloroplast genome sequences

**Fig. 2:**
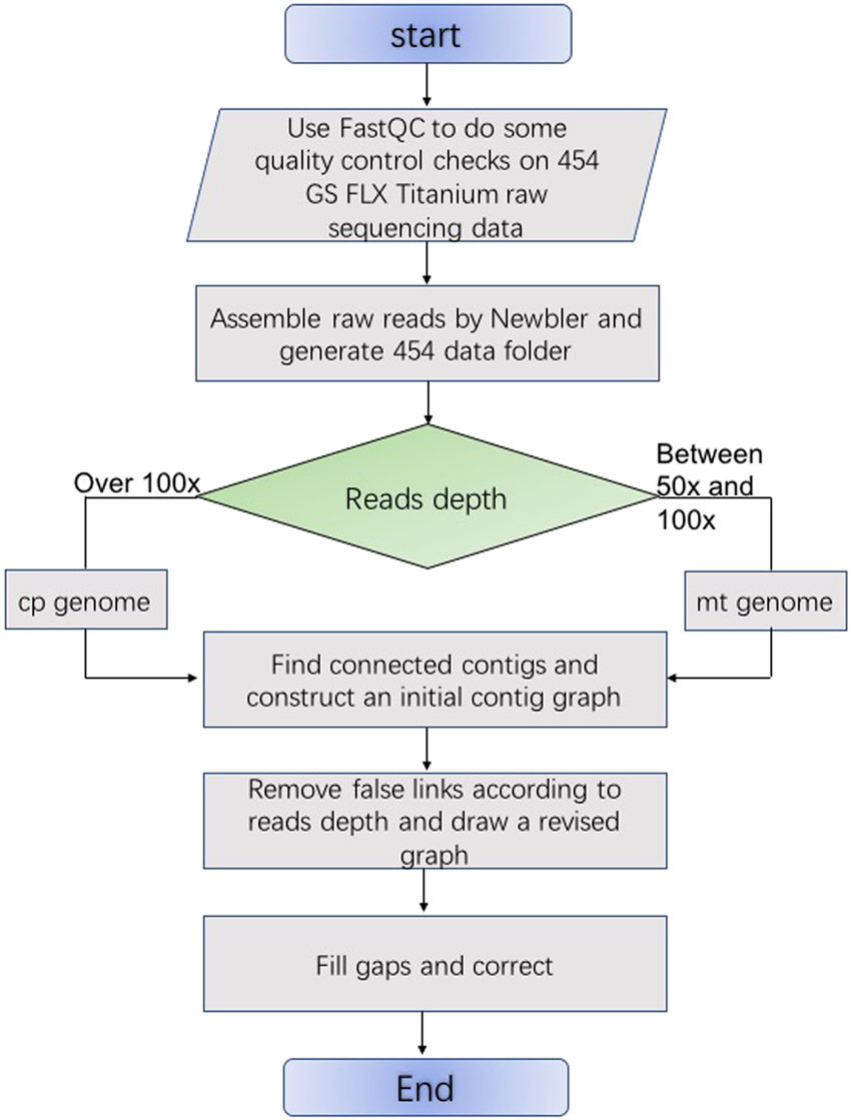
The flow chart of a novel method for organellar genome assembly. The assembly process includes data preparation, assembly of raw reads according to read depth of contigs, the creation of the contig graph, and the construction of the organellar genome sequences. The mitochondrial genome of *Ziziphus jujuba* and the organellar (mitochondrial and chloroplast) genomes of *Salix suchowensis* were assembled using this method

Two approaches for completing high quality organellar genome sequences from NGS data are shown in Figs. 1 and 2. The assembly process includes data preparation, assembly of raw reads according to read depth of contigs, the creation of the contig graph, and the construction of the organellar genome sequences. Unlike the traditional method, there is no need to isolate chloroplast or mitochondrial DNAs from a mixture of nuclear and organellar DNAs during the sample preparation.

### Data preparation

Whole-genome sequencing of an ornamental plant, *S. suchowensis*, was conducted on the Roche 454 and Illumina HiSeq 2000 sequencing systems at Nanjing Forestry University.

The fruit tree *Z. jujuba* was grown at Nanjing Forestry University, and its total DNA was extracted using a DNeasy Plant Mini kit^22^. The 454 pyrosequencing was performed on a 454 GS FLX Sequencer with XLR 70 Titanium kit (Roche Diagnostics) following the manufacturer’s standard protocol (Roche Diagnostics)^23^.

### Chloroplast genome assembly of *Z. jujuba*

The pipeline used for the assembly of the chloroplast genome of *Z. jujuba* is shown in Fig. 1. The chloroplast genomes of homologous species are similar and can be used as reference genomes to obtain the order of contigs. Sequencing reads from the Roche 454 system were initially mapped to land plant chloroplast genome sequences through BLASTN search^24^. Amos^25^, Minimus^26^, and Phrap software^27^ were then used to assemble the sequences. The detailed parameters for BLASTN were: *blastn –db database_name –query input_file –out output_file –evalue 1e-5 –word_size 9 –outfmt 6*.

Default parameters were used for Amos, Minimus, and Phrap. Connected contigs were linked, and the gaps were filled by BLASTN and PCR experiments. The whole-genome sequence was corrected based on Illumina sequencing data using BWA software^28^.

### A novel method for organellar genome assembly

A novel method for organellar genome assembly is shown in Fig. 2. Most chloroplast genomes are conserved and have a quadripartite organization, consisting of two copies of inverted repeats, a large-single-copy region, and a small single-copy region. The pipeline shown in Fig. 1 can be used to complete most chloroplast genome assemblies. However, assembling the mitochondrial genomes of related species by homology is more complicated, as reference genomes provide less information. Furthermore, the pipeline in Fig. 1 cannot determine the contig connection order. Thus, the method cannot fully complete the mitochondrial genome assembly. The pipeline shown in Fig. 2 can obtain the structural information and connect contigs easily.

The input of the procedure is the sequencing reads from the Roche 454 sequencing system. Newbler software was first used to assemble the raw reads and produce longer contigs. Mitochondrial and chloroplast genome-related contigs were then isolated from nuclear contigs. Contigs were divided into three categories: high read depth contigs, medium read depth contigs, and low read depth contigs. According to statistics from different plant species, high read depth contigs mainly belong to chloroplast genomes and nuclear repeat sequences, medium read depth contigs mainly belong to mitochondrial genomes and nuclear repeat sequences, and low read depth contigs belong to the nuclear genome. In this paper, we used read depth contigs over 100× as chloroplast genome candidate contigs and contigs between 50× and 100× as mitochondrial genome candidate contigs. Notably, the parameters for this step can be adjusted based on the user’s own sequencing data.

The mitochondrial genome of *Z. jujuba* and the organellar (mitochondrial and chloroplast) genomes of *S. suchowensis* were assembled. Organellar contig graphs were plotted through Perl scripts. A visualized map was constructed using OmniGraffle software^29^.

### Gap filling and correction

In our study, database indexing was used to fill the remaining gaps between sequences. As shown in Table 1, there are six steps for filling the remaining gaps. The input is related contigs with remaining gaps and the raw reads database. The first step is to prepare the query sequence with gaps. In the second step, we specified related options and searched the database using BLASTN to create a lookup table. The output format of the results can be adjusted through user options^30^. The third step is to discover matches between sequences and the database using BLASTN with an *E* value of 1e−5 ^30^. During this process, the position may not be located accurately, therefore this step should be iterated additional times. Finally, we assemble these alignments by the program Phrap^27^.

**Table 1:** Remaining gap filling

Procedure: gap filling
Input: related contigs with remaining gaps
Output: related contigs
Step 1: prepare the query sequence;
Step 2: “setup”, specify related options and database, create a lookup table;
Step 3: “BLASTN search”;
Step 4: “back-track”, input the preliminary matches and locate the insertions and deletions of uncertain sequences;
Step 5: output the results in a file;
Step 6: Phrap software assembles these alignments

The PCR experimental reagents for gap filling in the *Z. jujuba* chloroplast genome included 100 ng genomic DNA, 2 μl dNTP (2.5 Mm each), 2.5 μl 10× Ex Taq buffer (Mg^2+^ free), 0.25 μl Ex Taq DNA polymerase, 1.25 μl MgCl_2_ (25 Mm), 0.25 μl 0.1% BSA, and 1.25 μl of each primer (10 mmol/l). The amplification conditions were 94 °C for 5 min, followed by 30 cycles of 94 °C for 30 s, 58 °C for 30 s, and 72 °C for 10 min. Different primers had different annealing temperatures, which varied from 56 °C to 60 °C^22^.

After obtaining a reference genome, shorter reads from Illumina sequencing platform are mapped to reference genomes through BWA^31^, forming a consensus sequence to determine whether there are base differences in the reference genome.

The detailed procedure of aligning Illumina short reads against the reference genomes using BWA are as follows:

1. build **index**: bwa index –a bwtsw reference.fa
2. find **SA coordinates**: bwa aln –t 30 –f single.sai reference.fa single.fastq
3. convert **SA coordinates** and output **sam**: bwa samse –f single.sam reference.fa single.sai single.fastq
4. convert **sam** to **bam**: samtools view –bS single.sam > single.bam
5. extract results that can align to the reference sequence: samtools view –Bf 4 single.bam > single.F.bam
6. **bam** to **fastq**: bam2fastq single.F.bam –o single.fq
7. assembly: runAssembly –cpu 10 –het –sio –m –urt –large –o result single.fq.

The process of alignment allows for 1–2 bases error, and after these steps, we can identify and correct the reference sequences.

PCR experiments have verified that this method can effectively correct errors in the assembled genome^22^.

### Organellar genome analysis

To identify the phylogenetic position of horticultural plants, 39 horticultural plant mitochondrial genomes were downloaded from NCBI. A phylogenetic tree was constructed based on 15 protein-coding genes (*atp1, atp9, ccmB, cob, cox1, cox3, nad1, nad3, nad4, nad4L, nad6, nad7, nad9, rps3*, and *rps4*). The sequences of these genes were extracted by local Perl scripts. The program MEGA^32^ was used for the alignment of conserved genes, building a tree of the species, and calculating GC content^32^. MEGA integrates multiple functions including aligning multiple sequences by ClustalW and the algorithms of neighbor-joining (NJ), maximum likelihood (ML), and minimum evolution (ME). The alignment of conserved genes was modified manually to remove gaps.

## Results

### Sequencing data

The sequencing reads of *Z. jujuba* were generated using the Roche 454 GS FLX sequencer. A total of 573,141 raw reads were obtained with a mean length of 360 bp. After the quality checking by the program FastQC^33^, we retained 70,931 sequences (~34.50 Mb) and 2950 contigs whose quality was acceptable^22^. The sequencing of *S. suchowensis* was performed on the Roche 454 and Illumina HiSeq 2000 systems. A total of 1,240,387 raw reads were produced with a total length of 702,204,081 bp, and the mean size was 567 bp. After checking quality by FastQC^33^, we retained 235,005 contigs, and the longest length of a contig was 349,758 bp.

### Complete chloroplast genome

**Fig. 3:**
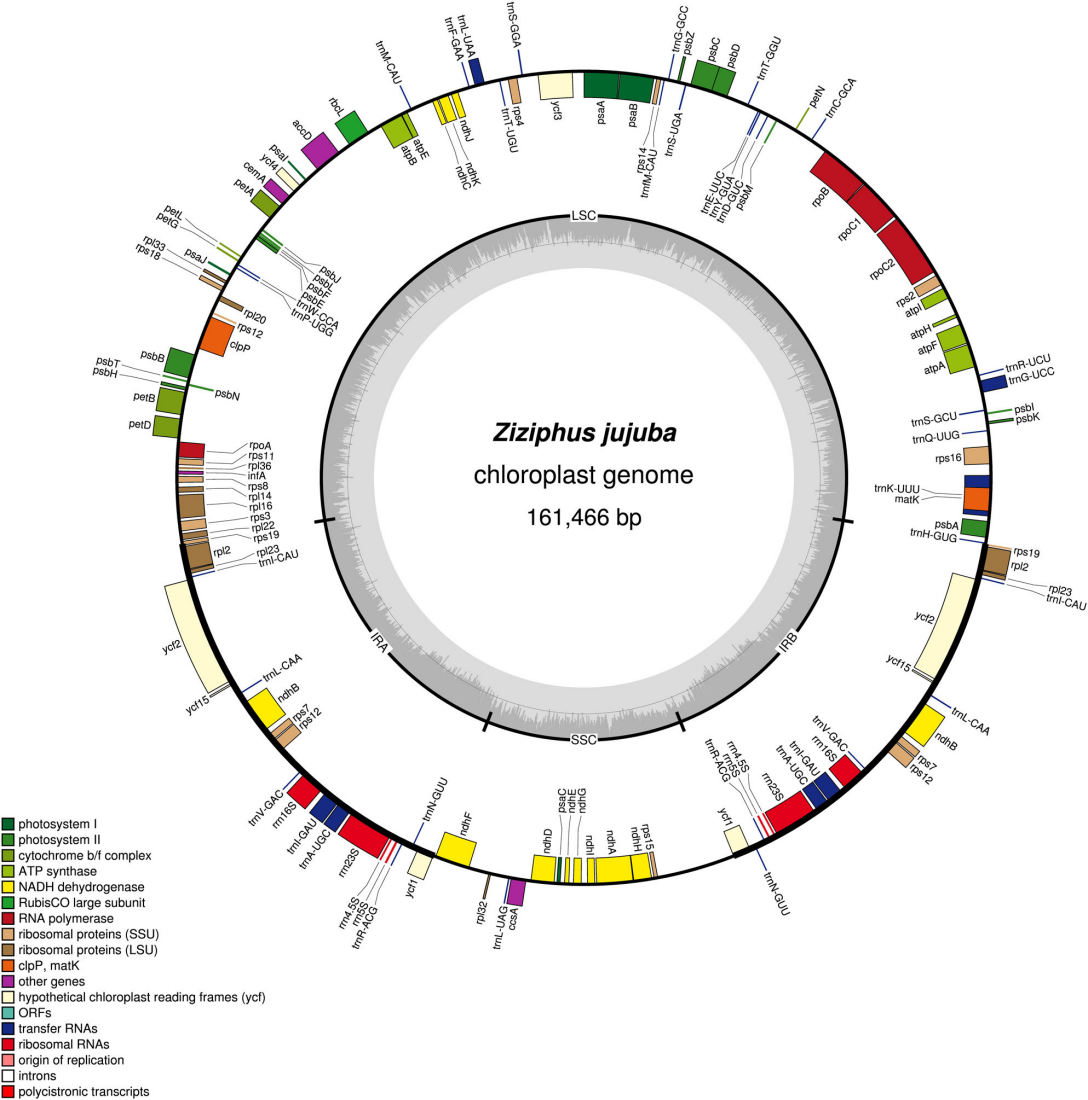
*Ziziphus jujuba* circular chloroplast genome map. Genes that belong to different functional groups are color-coded. GC content is represented on the inner circle by the dark gray plot. *Ziziphus jujuba* contained 112 unique genes, including 78 protein-coding genes, 30 tRNA genes, and 4 rRNA genes

The Amos^20^ and Minimus software^34^ with default parameters were used to assemble the chloroplast genome sequences of *Z. jujuba* (shown in Fig. 3). The sequences and detail information of each contig were stored in a fasta formatted file called “Contigs.fasta” and a text file called “Contigs.contig”, respectively. In this process, 70,931 sequences (~34.50 Mb) and 2950 contigs were assembled. We further obtained 62 contigs by Phrap software^27^ with default parameters. To confirm the location of the contigs in the *Z. jujuba* chloroplast genome, the final contigs were mapped to the *Arabidopsis thaliana* chloroplast genome. The N50 of contigs and the percentage of the organellar genome covered by the contigs of *Z. jujuba* were 84,718 bp and 98.38%, respectively.

Two methods, BLASTN search and PCR amplification with Sanger sequencing, were used to fill the remaining gaps. The gaps were assembled by Phrap^24^. We filled 2611 bp gaps completely by BLASTN and PCR for the *Z. jujuba* chloroplast genome. The problem of tandem bases in Roche 454 sequencing data may have an influence on the assembly accuracy^35^. To obtain a high quality chloroplast genome, the assembled sequences were corrected based on high quality Illumina sequencing data by using BWA software. The Illumina sequencer could produce reads with high accuracy^36^. In this process, we successfully corrected 165 errors in the complete mitochondrial genome of *S. suchowensis*.

**Fig. 4:**
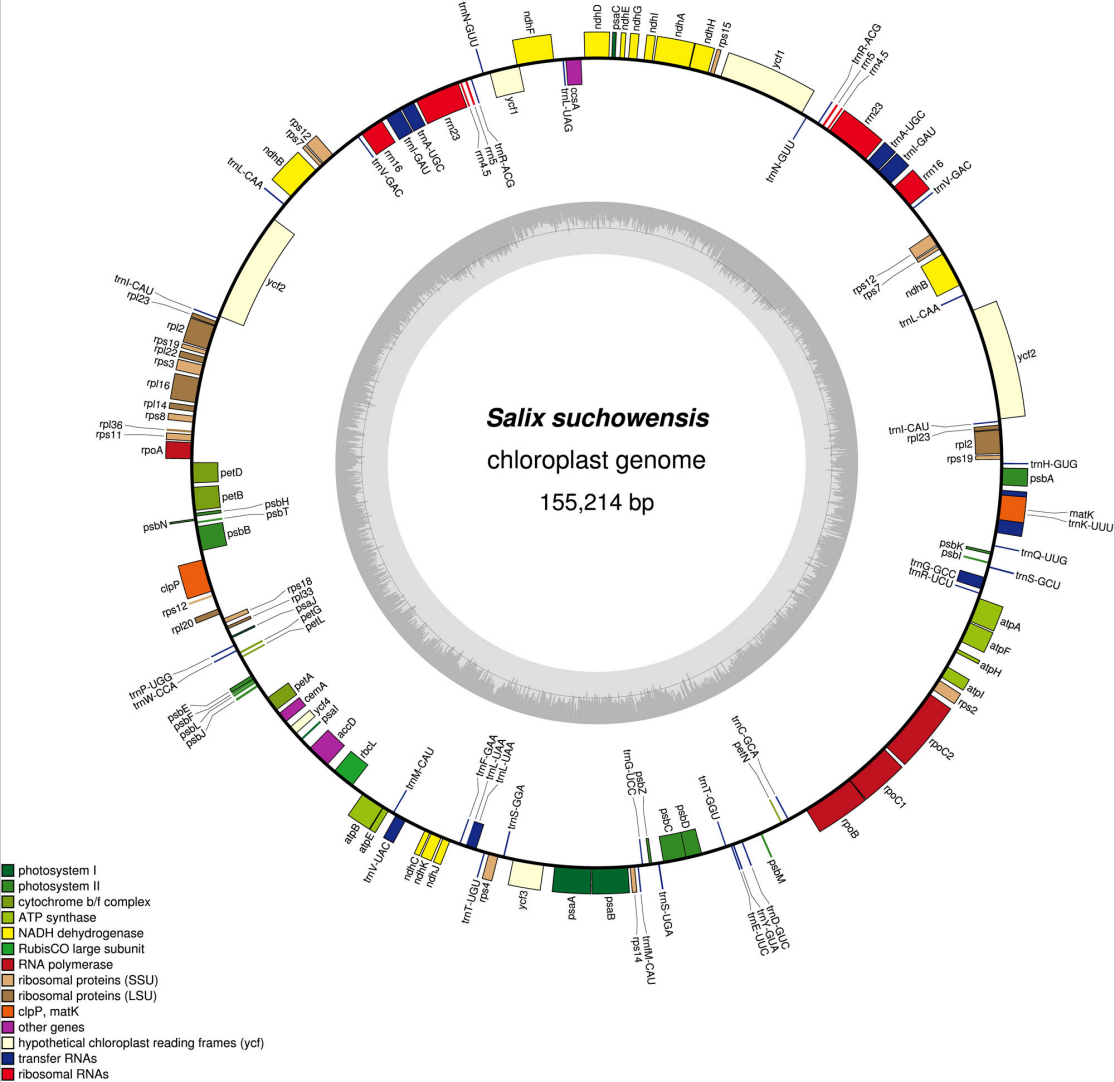
*Salix suchowensis* circular chloroplast genome map. Genes that belong to different functional groups are color-coded. GC content is represented on the inner circle by the dark gray plot. A total of 110 unique genes were annotated, including 76 protein-coding genes, 30 tRNA genes, and 4 rRNA genes

The chloroplast genome of *S. suchowensis* (Fig. 4) was assembled using the novel approach shown in Fig. 2. For *S. suchowensis* (NC_029317.1), 1,240,387 raw reads with a total length of 702,204,081 bp were first input into Newbler. Newbler software was used to assemble the Roche 454 GS FLX sequencing shorter reads and to produce contigs with longer length^37^. A contig graph was also plotted, in which the nodes are contigs and the edges are the reads spanning them. All the information on this graph, except the actual read alignments and consensus contig, is included in the 454 ContigGraph.txt file. There are several sections in the file. The first section is contig statistics, including contig number, name length, and contig read depth. The second section is the edge information, including the letter “C”, the contig number on the left end of the edge, 5′ or 3′ to indicate which end of the contig the left edge refers to, the contig number at the right end of the edge, 5′ or 3′ to indicate which end of the contig the right edge refers to, and the depth of the edge (Table 2). The first and second sections were used to assemble the organellar genomes. After calculation, we obtained 235,005 contigs, of which the longest contig was 349,730 bp. The chloroplast genome of *S. suchowensis* has been submitted to http://bio.njfu.edu.cn/gb2/gbrowse/Salix_su_cp_sun/.

**Table 2:** Representative example of 454ContigGraph.txt file

	Contig number		Name length		Contig read depth
1	contig00001		349,730		22.3
	Contig number (left)	5′ or 3′	Contig number (right)	5′ or 3′	Edge depth
C	8	5′	13	3′	588

### Complete mitochondrial genomes

**Fig. 5:**
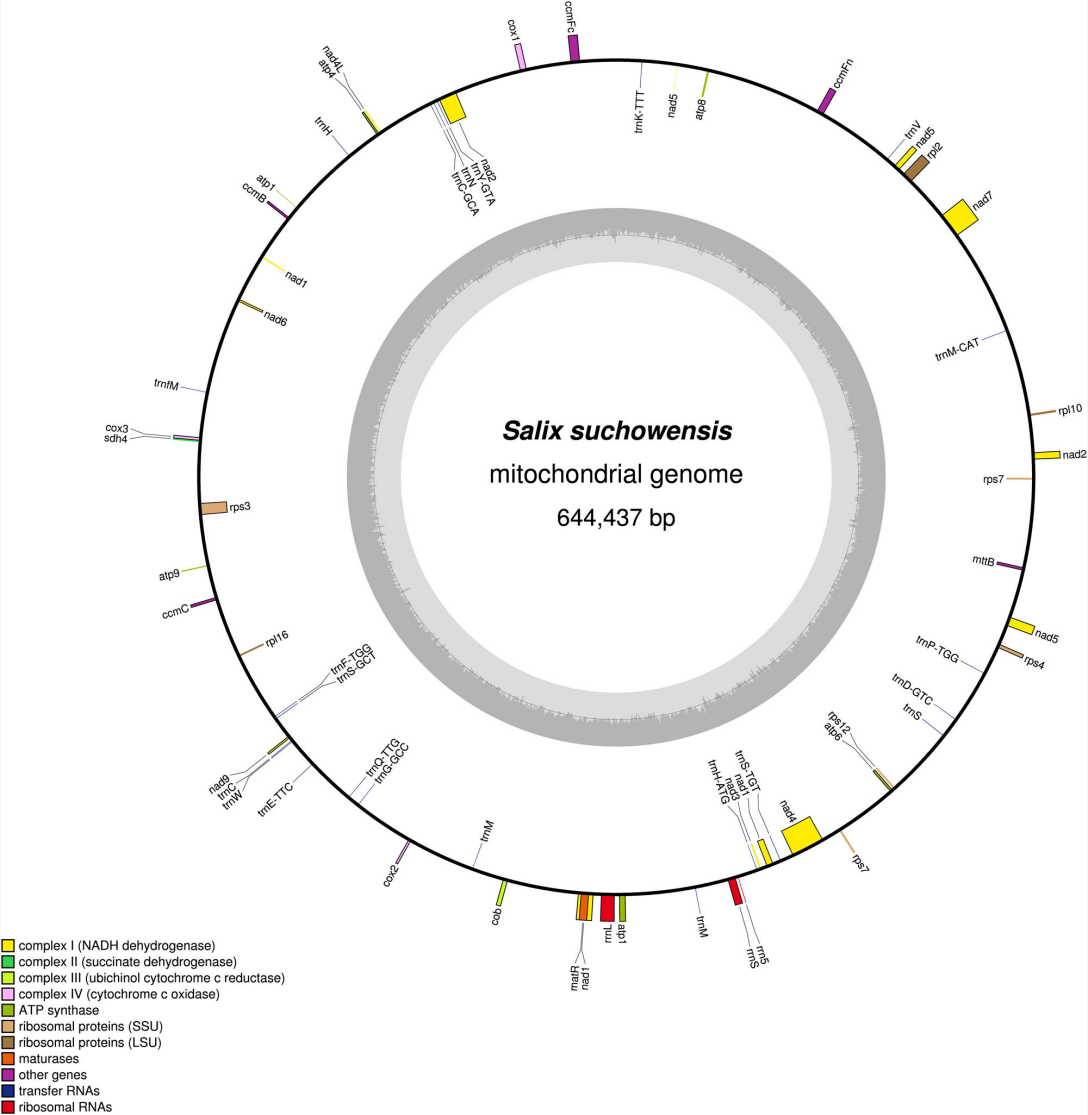
The circular mitochondrial genome of *Salix suchowensis*. Genes belonging to different functional groups are color-coded. GC content is represented on the inner circle by the dark gray plot. This long circular mitochondrial genome encodes 58 unique genes (32 protein-coding genes, 23 tRNA genes, and 3 rRNA genes), and 9 of the 32 protein-coding genes contain 17 introns

**Fig. 6:**
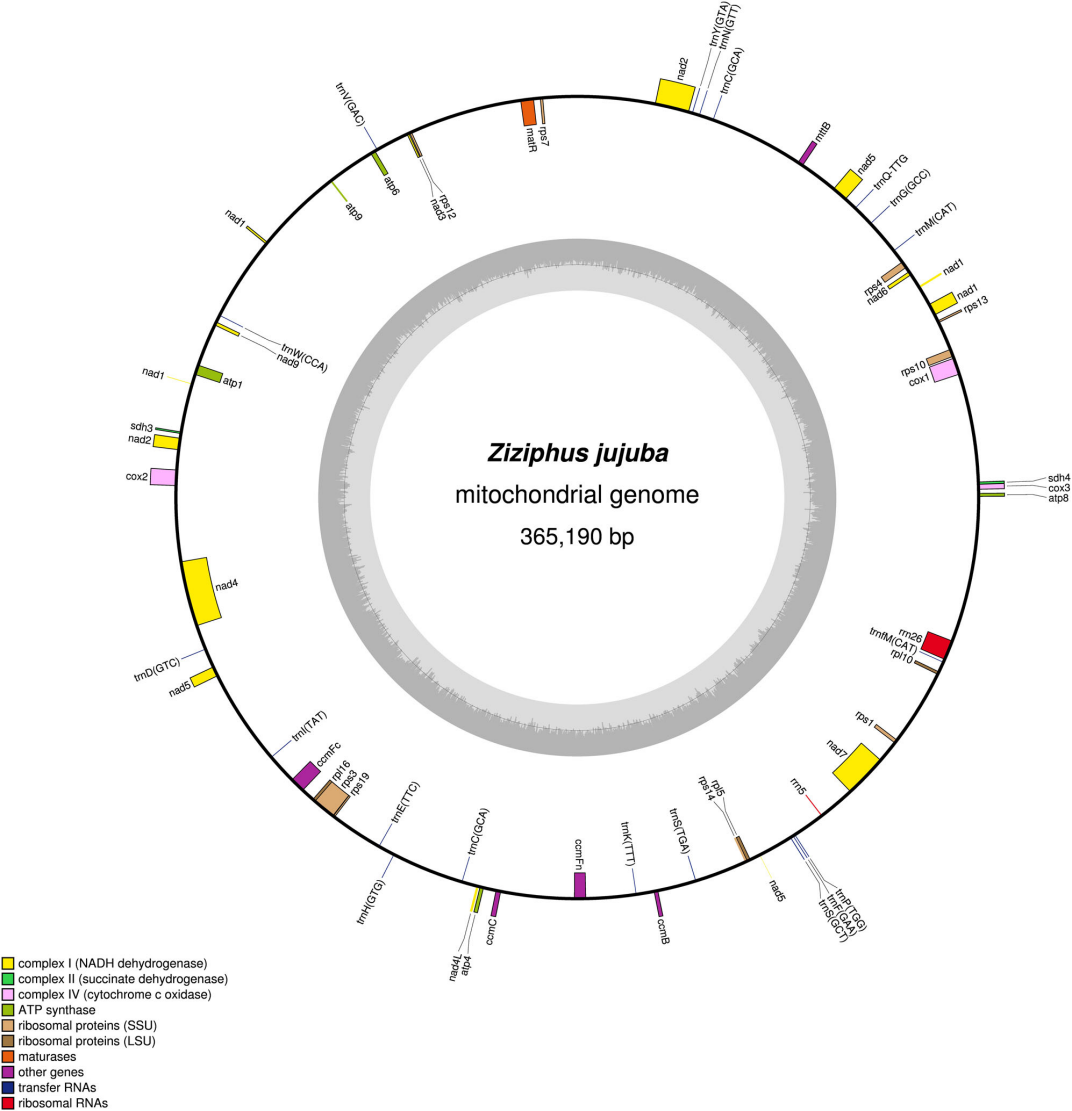
The circular mitochondrial genome of *Ziziphus jujuba*. Genes belonging to different functional groups are color-coded. GC content is represented on the inner circle by the dark gray plot. This long circular mitochondrial genome encodes 58 unique genes (37 protein-coding genes, 19 tRNA genes, and 2 rRNA genes)

Our previous study showed that the contig read depths in the nuclear DNA, mitochondrial, and chloroplast DNA were ~1–2×, 50–100×, and over 100×, respectively^38^. According to read depth, we filtered out mitochondrial contigs that contained essential mitochondrial genes for further assembly. An initial mitochondrial contig graph was then constructed by Perl scripts based on the file 454ContigGraph.txt. In this process, the contigs in the first row of the file were used as a starting point to transverse all adjacent contigs; if there was a breakpoint, a new contig was selected to repeat the process. Contigs already connected with the original seed were considered as new seeds for searching its connected contigs recursively. In addition, because of the high frequency of chloroplast genomic DNA in the mitochondrial genome^39^, chloroplast-like contigs that were partially in a path were also saved for further analysis. At the same time, false links and forks that might belong to different genomes were removed according to the read depths of the contigs. A revised graph with repetitive contigs was constructed and is shown in Fig. S1. Eventually, a high-quality mitochondrial genome including 13 contigs with a total length of 644,437 bp was completed^40^ (Fig. 5). Similarly, we successfully assembled the mitochondrial genome of *Z. jujuba* and submitted it to the NCBI Genome database (NC_029809.1). The circular mitochondrial genome of *Z. jujuba* is shown in Fig. 6.

### Analysis of organellar genomes

Chloroplasts and mitochondria are thought to have been developed during the formation of membrane compartments in eukaryotic cells in evolution. Nevertheless, some studies of their gene organization and content indicate that chloroplasts and mitochondria originated from cyanobacteria and alpha-proteobacteria, respectively^41^. Mitochondrial genome size, genome reorganization, and number of genes transferred from chloroplast genome into mitochondrial genome show a notable difference among higher plants because of homologous recombination during the evolution of the mitochondrial genome. Therefore, it is difficult to detect mitochondrial ancestry^42^.

Organellar genome analysis indicated that all 39 horticultural plants have similar mitochondrial and chloroplast sequence organization to most species. The average length of the mitochondrial genomes of these plants is 500,348 bp. In general, the base content of the *S. suchowensis* mitochondrial genome is A (27.43%), T (27.59%), C (22.34%), G (22.64%), and the base content of the *Z. jujuba* mitochondrial genome is A (27.32%), T (27.41%), C (22.92%), G (22.35%). Similar to that in most horticultural plants (Table S2), a slight bias toward A+T rich features was shown in the mitochondrial genomes of these two plants.

The chloroplast genomes of *Beta macrocarpa*, *Butomus umbellatus*, *Cucurbita pepo*, *Malus domestica*, and *Vaccinium macrocarpon* have not been included in NCBI. The average length of the completed chloroplast genomes of the 34 remaining horticultural plants is 151,720 bp. Among of them, *Nelumbo nucifera* has the longest length at 163,330 bp and *Welwitschia mirabilis* has the shortest length at 119,726 bp. Similar to mitochondrial genomes, in horticultural plants, A+T bases occupy a large proportion of the chloroplast genomes (Table S3).

**Fig. 7:**
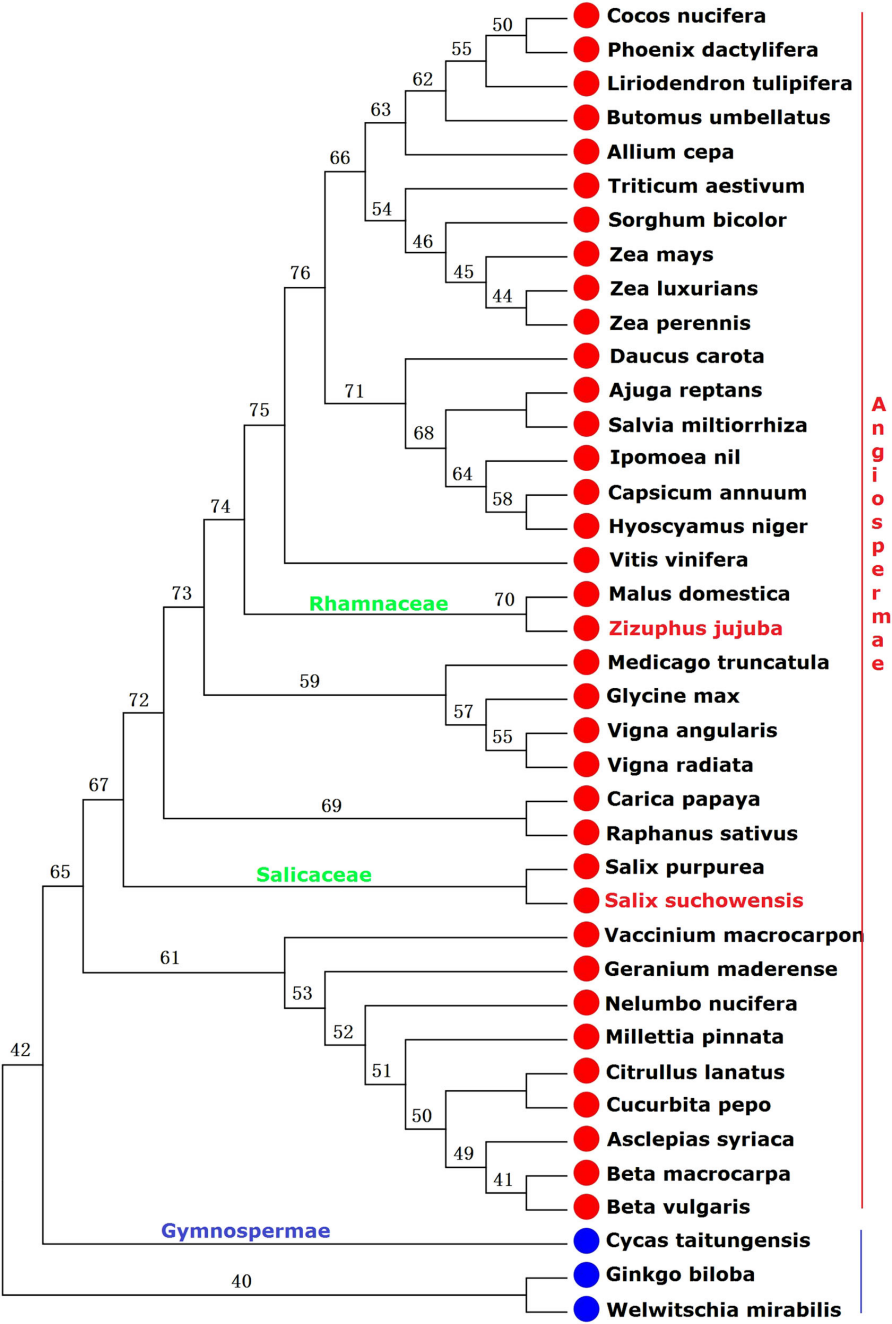
The neighbor-joining tree was constructed based on 15 conserved protein-coding genes of 39 horticultural plant mitochondrial genomes. The red and blue represent the categories angiospermae and gymnospermae, respectively. The numbers at the nodes are bootstrap support values

Phylogenetic analysis of complete organellar genomes can identify plant evolutionary relationships accurately. In this study, a phylogenetic tree was constructed by an alignment of 15 protein-coding genes from 39 horticultural plants. As illustrated in Fig. 7, the 39 horticultural plants were categorized into two major groups: gymnospermae (colored by blue) and angiospermae (colored by red). The phylogenetic tree supported the separation of angiospermae and gymnospermae with 65% bootstrap value. A total of 27 dicotyledons in these plants were grouped in the category of angiospermae. The bootstrap value for the separation of eudicots and monocots is 66%. According to the phylogenetic tree, *Z. jujuba* is evolutionally closer to *Malus domestica* than to other plants. The sister relationship between *S. suchowensis* and *S. purpurea* is strongly supported^43^.

In plant evolution, the number of protein-coding genes in mitochondrial genomes declines (Table S1). As a representative species of dicot, the mitochondrial genome of *Vitis vinifera* has 61 protein-coding genes, which is almost the maximum number for all horticultural plants. Protein-coding genes such as *PetA* and *Ycf4* in the mitochondrial genome of *Vitis vinifera* have been horizontally transferred from chloroplast DNA. In contrast, the mitochondrial genome of *Geranium maderense*, *Allium cepa*, and *Vigna angularis* have the minimum number of protein-coding genes: 27, 26, and 25 respectively. Succinate dehydrogenase genes are missing in *Ajuga reptans* and 18 other species such as *Medicago truncatula*. Most of the 39 horticultural plants had lost the *rps11* gene. *MttB*, which encodes a transport membrane protein, was lost in *Beta macrocarpa* and *V. angularis*. More unusually, contrasting with three species in gymnospermae, the protein-coding genes of *S. suchowensis* and *Z. jujuba* include the same ATP synthesis genes (*Atp1, Atp4, Atp6, Atp8*, and *Atp9*) and NADH dehydrogenase subunits (*Nad1, Nad2, Nad3, Nad4, Nad4L, Nad5, Nad6, Nad7*, and *Nad9*). However, all three plants of the gymnospermae have lost *rpl10*, and thus, it can be inferred that *rpl10* has gradually developed into a pseudogene during the evolution of gymnosperms.

Some tRNA genes from chloroplast genomes have been inserted into mitochondrial genomes through intercellular transfers^39^. Our data show that the chloroplast genes *trnM, trnH*, and *trnS* are found in *S. suchowensis* and *Z. jujube*. The same gene insertion event was observed in other 28 horticultural species. The tRNA gene transformation of these plants may indicate that this phenomenon occurred before the formation of angiosperms. In addition, a mitochondrial-like gene, *trnE*, is found in many plants, with the exception of *Cocos nucifera* and *Ginkgo biloba*. All horticultural species can be generally separated into two groups according to their types of ribosomal genes. One group has the rRNA *genes rrn5, rrn18*, and *rrn26*, including *Ajuga reptans*, and the other group has the genes *rrn5*,* rrnL*, and *rrnS*, including *G. maderense*. Two plants are considered the exceptions: *Z. jujuba* lacks the *rrn18* gene, and *B. umbellatus* has rrn16 in its mitochondrial genome.

## Discussion

In this paper, we proposed an innovative and efficient assembly approach (shown in Fig. 2) for organellar genome assembly of horticultural plants using next generation sequencing data without isolating organellar DNA. We assembled the mitochondrial genome of *Z. jujuba* and the mitochondrial and chloroplast genomes of *S. suchowensis* using this pipeline. This study proved that our method can assemble both chloroplast and mitochondrial genomes.

Compared to other sequencing platforms such as SOLiD^44^ and Illumina HiSeq^36^, Roche 454 sequencing is a high-throughput and low-cost sequencing technology, which can produce longer and relatively accurate reads (Table 3). In addition, a single lane of the Roche 454 platform is sufficient for organellar genome assembly^37^. Chloroplast or mitochondrial sequences can be well separated based on the read depths of the contigs derived from the sequencing reads.

**Table 3:** Advantages/disadvantages of different sequencing technologies

Sequencing technology	Advantages	Disadvantages
454 GS FLX	1. A single lane is enough for organelle-rich samples;	1. Out of date, less technical support at present;
	2. Accuracy is above 99%, many data in NCBI have not been effectively used, especially for assembling mitochondrial and chloroplast genomes;	2. Homopolymers, long stretches of the same base, such as AAA or GGG, may cause errors. Thus, insertion–deletion is the main error type, rather than substitution
	3. Low data and low cost can complete mitochondrial and chloroplast genome assembly	
	4. Connections between contigs are clear;	
	5. Only need 2 to 3 times the data for whole-genome sequencing	
Illumina HiSeq	Produces a larger number of high precision sequencing reads	Its short reads can barely be used to assemble chloroplast genomes of plants and animal mitochondrial genomes through reference genomes. However, it is difficult to assemble the mitochondrial genome of plants with a complex structure using the Illumina platform
Third generation sequencing	Much longer reads, and the connections between contigs are clear	1. Error rate of a single read goes up to 15–40%;
		2. Whole-genome sequencing requires 30× to 70× coverage;
		3. High cost
PCR	Highly precise sequencing reads can assemble chloroplast genomes of plants and animal mitochondrial genomes	Unable to complete mitochondrial genome assembly of plants with a complex structure

To ensure high assembly quality, some quality control steps were included in this study. First, FastQC was used to check the raw sequence reads, which can provide a global picture of the quality of the sequencing data. Second, if the same species had both 454 sequencing data and Illumina data, Illumina sequencing data can be used for the correction of its organellar genome assembly using BWA. PCR experiments have proved that the BWA-based method can efficiently correct genome assemblies^22^.

After obtaining the complete organellar genomes of horticultural plants, related genes, including protein genes, tRNAs, and rRNAs, were identified subsequently. GC content was also analyzed by a Perl script. Repeat sequences can be detected, which provide useful information to characterize mitochondrial genomes^45^, to investigate the influence of repeat sequences on mitochondrial genome size and to identify evolutionary changes in mitochondrial genome organization and structure^46,47^.

In the process of evolution, mitochondria and chloroplast have a prokaryotic ancestry that could be suggested by their functions and genome organizations^48^. Moreover, most activities of the mitochondrial and chloroplast genomes are occasional and have an immediate or delayed impact on nuclear genome evolution because the nuclear genome and organellar genomes work together^48^. As a result, complete organellar genomes provide important to support breeding projects^49^ and a better understanding of DNA transfers within and between the genomes and genomic recombination, which will facilitate the biological studies of horticultural plants in the future^21^.

## Conclusions

In this paper, we have successfully applied a new, efficient approach to determine the complete chloroplast and mitochondrial genomes of two horticultural plants from Roche 454 GS FLX sequencing data. The Roche 454 GS FLX sequencer could generate longer sequencing reads^37^. Newbler, an efficient assembly software, also enabled the organellar genome assembly with high quality^50^. The read depths of contigs in the chloroplast and mitochondrial genomes rely on the proportion of total DNA and their copy numbers in the cell^37^. According to the read depths of the contigs and the copy numbers of the organellar genomes, we assembled chloroplast and mitochondrial DNA from the NGS data. Unlike the traditional method, there is no requirement to isolate organellar DNAs from total DNAs. Our method can also be extended to other platforms. We believe that this approach can be used for organellar genome assembly in other horticultural plants. Our method can also be applied to evaluate other sequencing platforms^51^.

A comparative analysis of the mitochondrial and chloroplast genomes of horticultural plants shows that they share most common genomic features with other plants. Mitochondrial gene comparison with other horticultural species will contribute to a systemic understanding of plant evolution. Complete horticultural organellar genomes and a phylogenetic analysis of these organellar genomes would provide useful clues for better understanding intra-genomic and inter-genomic DNA transfers and genomic recombination in horticultural plants^21^.

## Electronic supplementary material

Table S1

Table S2

Table S3

Figure S1

Sequences of contigs extraction

GC Content Analyzation

Newbler Assembly

Contigs Selection

Contigs Connection

## References

[1] Richman, V., Bennett, J., Jackson, R.S. et al. Horticulture- Plant needs, Horticultural plants. *Science Encyclopedia*. Web. 20 Dec 2017. http://science.jrank.org/pages/3392/Horticulture.html.

[2] Pyke KA (1999). Plastid division and development. Plant Cell.

[3] Mccoy SR, Kuehl JV, Boore JL, Raubeson LA (2008). The complete plastid genome sequence of Welwitschia mirabilis: an unusually compact plastome with accelerated divergence rates. BMC Evol. Biol..

[4] Wu, C. S., Wang, Y. N., Liu, S. M., & Chaw, S. M. Chloroplast Genome (cpDNA) of *Cycas taitungensis* and 56 cp protein-coding genes of *Gnetum parvifolium*: insights into cpDNA evolution and phylogeny of extant seed plants. *Mol. Biol. Evol.* **24**, 1366–1379 (2007). 10.1093/molbev/msm059 17383970

[5] Yang M (2012). The complete chloroplast genome sequence of date palm (*Phoenix dactylifera* L.). PLoS ONE.

[6] Kubo T, Newton KJ (2008). Angiosperm mitochondrial genomes and mutations. Mitochondrion.

[7] Tanaka Y, Tsuda M, Yasumoto K, Yamagishi H, Terachi T (2012). A complete mitochondrial genome sequence of Ogura-type male-sterile cytoplasm and its comparative analysis with that of normal cytoplasm in radish (*Raphanus sativus* L.). BMC Genom..

[8] Alverson AJ (2010). Insights into the evolution of mitochondrial genome size from complete sequences of *Citrullus lanatus* and *Cucurbita pepo* (Cucurbitaceae). Mol. Biol. Evol..

[9] Alverson AJ, Zhuo S, Rice DW, Sloan DB, Palmer JD (2011). The mitochondrial genome of the legume *Vigna radiata* and the analysis of recombination across short mitochondrial repeats. PLoS ONE.

[10] Jeong YM (2014). The complete mitochondrial genome of cultivated radish WK10039 (*Raphanus sativus* L.). Mitochondrial DNA A DNA Mapp. Seq. Anal..

[11] Alverson AJ (2010). Insights into the evolution of mitochondrial genome size from complete sequences of *Citrullus lanatus* and *Cucurbita pepo* (Cucurbitaceae). Mol. Biol. Evol..

[12] Atherton RA (2010). Whole genome sequencing of enriched chloroplast DNA using the Illumina GAII platform. Plant Methods.

[13] Unseld M, Marienfeld JR, Brandt P, Brennicke A (1997). The mitochondrial genome of *Arabidopsis thaliana* contains 57 genes in 366,924 nucleotides. Nat. Genet..

[14] Handa H (2003). The complete nucleotide sequence and RNA editing content of the mitochondrial genome of rapeseed (*Brassica napus* L.): comparative analysis of the mitochondrial genomes of rapeseed and *Arabidopsis thaliana*. Nucleic Acids Res..

[15] Ogihara Y (2005). Structural dynamics of cereal mitochondrial genomes as revealed by complete nucleotide sequencing of the wheat mitochondrial genome. Nucleic Acids Res..

[16] Jansen RK (2010). Methods for obtaining and analyzing whole chloroplast genome sequences. Methods Enzymol..

[17] Cronn R (2008). Multiplex sequencing of plant chloroplast genomes using Solexa sequencing-by-synthesis technology. Nucleic Acids Res..

[18] Moore MJ (2006). Rapid and accurate pyrosequencing of angiosperm plastid genomes. BMC Plant. Biol..

[19] Tangphatsornruang S (2010). The chloroplast genome sequence of mungbean (*Vigna radiata*) determined by high-throughput pyrosequencing: structural organization and phylogenetic relationships. DNA Res..

[20] Kumar S, Stecher G, Tamura K (2016). MEGA7: molecular evolutionary genetics analysis version 7.0 for bigger datasets. Mol. Biol. Evol..

[21] Simon PW (2012). *De novo* assembly and characterization of the carrot mitochondrial genome using next generation sequencing data from whole genomic DNA provides first evidence of DNA transfer into an angiosperm plastid genome. BMC Plant Biol..

[22] Ma Q (2017). Complete chloroplast genome sequence of a major economic species, *Ziziphus jujuba* (Rhamnaceae). Curr. Genet..

[23] Ma Q (2014). Identification and characterization of nucleotide variations in the genome of *Ziziphus jujuba* (Rhamnaceae) by next generation sequencing. Mol. Biol. Rep..

[24] Camacho C. et al. BLAST plus: architecture and applications. *BMC Bioinformatics* **10**, 421 (2009). 10.1186/1471-2105-10-421 PMC2803857 20003500

[25] Treangen TJ, Sommer DD, Angly FE, Sergey K, Mihai P (2011). Next generation sequence assembly with AMOS. Curr. Protoc. Bioinformatics.

[26] Sommer DD, Delcher AL, Salzberg SL, Pop M (2007). Minimus: a fast, lightweight genome assembler. BMC Bioinformatics.

[27] Ewing B, Green P (1998). Base-calling of automated sequencer traces using Phred. II error probabilities. Genome Res..

[28] Peters, D., Qiu, K., Liang, P. Faster short DNA sequence alignment with parallel BWA. *AIP Conf. Proc.* **1368**, 131–134 (2011).

[29] Surhone, L. M., Tennoe, M. T., Henssonow, S. F., Group, T. O., & Done, G. T. *OmniGraffle* (Betascript Publishing, Beau Bassin, Mauritius, 2010).

[30] Zhao K, Chu X (2014). G-BLASTN: accelerating nucleotide alignment by graphics processors. Bioinformatics.

[31] Li, H. Aligning sequence reads, clone sequences and assembly contigs with BWA-MEM. Preprint at https://arxiv.org/pdf/1303.3997.pdf (2013).

[32] Tamura K, Stecher G, Peterson D, Filipski A, Kumar S (2013). MEGA6: molecular evolutionary genetics analysis version 6.0. Mol. Biol. Evol..

[33] Andrews, S. FastQC: a quality control for high throughout sequence data. http://www.bioinformatics.babraham.ac.uk/projects/fastqc (2010).

[34] Sommer DD, Delcher AL, Salzberg SL, Pop M (2007). Minimus: a fast, lightweight genome assembler. BMC Bioinformatics.

[35] Shao W (2013). Analysis of 454 sequencing error rate, error sources, and artifact recombination for detection of low-frequency drug resistance mutations in HIV-1 DNA. Retrovirology.

[36] Nock CJ (2011). Chloroplast genome sequences from total DNA for plant identification. Plant Biotechnol. J..

[37] Zhang T, Zhang X, Hu S, Yu J (2011). An efficient procedure for plant organellar genome assembly, based on whole genome data from the 454 GS FLX sequencing platform. Plant Methods.

[38] Xuelin, W. et al. The whole genome assembly and comparative genomic research of *Thellungiella parvula* (*Extremophile crucifer*) mitochondrion. *Int. J. Genomics* **2016**, 5283628 (2016). 10.1155/2016/5283628 PMC4842374 27148547

[39] Wang D (2007). Transfer of chloroplast genomic DNA to mitochondrial genome occurred at least 300 MYA. Mol. Biol. Evol..

[40] Ye N (2017). Assembly and comparative analysis of complete mitochondrial genome sequence of an economic plant *Salix suchowensis*. Peer J..

[41] Barbrook AC, Howe CJ, Kurniawan DP, Tarr SJ (2010). Organization and expression of organellar genomes. Philos. Trans. R. Soc. B Biol. Sci..

[42] Ohyama K (2009). Gene content, organization and molecular evolution of plant organellar genomes and sex chromosomes: insights from the case of the liverwort *Marchantia polymorpha*. Proc. Jpn. Acad..

[43] Wei S (2016). Assembly and analysis of the complete *Salix purpurea* L. (Salicaceae) mitochondrial genome sequence. Springerplus.

[44] Wang W, Messing J (2011). High-throughput sequencing of three Lemnoideae (duckweeds) chloroplast genomes from total DNA. PLoS ONE.

[45] Knoop V., Volkmar U., Hecht J., & Grewe F. *Mitochondrial Genome Evolution in the Plant Lineage* 3–29 (Springer, New York, 2011).

[46] Etminan M, Fitzgerald JM, Gleave M, Chambers K (2010). Recombination and the maintenance of plant organelle genome stability. N. Phytol..

[47] Alverson AJ, Rice DW, Dickinson S, Barry K, Palmer JD (2011). Origins and recombination of the bacterial-sized multichromosomal mitochondrial genome of cucumber. Plant Cell.

[48] Chaubey, A. & Rajam, M. V. in *Plant Biology and Biotechnology* (eds Bahadur B., Venkat Rajam M., Sahijram L., Krishnamurthy K.) 179–204 (Springer, New Delhi, 2015).

[49] Peace CP (2017). DNA-informed breeding of rosaceous crops: promises, progress and prospects. Hortic. Res..

[50] Nederbragt AJ (2014). On the middle ground between open source and commercial software—the case of the Newbler program. Genome Biol..

[51] Greene CS, Troyanskaya OG (2012). Accurate evaluation and analysis of functional genomics data and methods. Ann. N. Y. Acad. Sci..

