# Supplementary material for: Organellar genome assembly methods and comparative analysis of horticultural plants
Source: Hortic Res. 2018 Jan 10;5:3. doi: 10.1038/s41438-017-0002-1 (PMC5798811; doi:10.1038/s41438-017-0002-1)
Supplement: Supplementary file 1 — Table S1 [file 41438_2017_2_MOESM1_ESM.docx]

Table S1 Gene profile and organization of the 39 horticultural mitochondrial genome

|  | Species | Gene | tRNA | rRNA |
| --- | --- | --- | --- | --- |
| 1 | Ajuga reptans | Atp1,Atp4,Atp6,Atp8,Atp9,Nad1,Nad2,Nad3,Nad4,Nad4L,Nad5,Nad6,Nad7,Nad9,Rps3,Rps4,Rps12,Rps13,Rpl23,Rpl2,Cox1,Cox2,Cox3,Cob,CcmB,CcmC,CcmFc,CcmFn,MatR,MttB | tRNA-Asp, tRNA-Met, tRNA-Ser, tRNA-Tyr, tRNA-Asn, tRNA-Cys, tRNA-Glu, tRNA-Gln, tRNA-Ile, tRNA-Pro, tRNA-Phe, tRNA-Trp | Rrn26, Rrn18, Rrn5 |
| 2 | Allium cepa | Atp1,Atp4,Atp6,Atp8,Atp9,Cox2,Cox3,Nad1,Nad2, Nad3,Nad4,Nad4L,Nad5,Nad6,Nad7,Nad9,Rps12,Cob,Cox2,CcmB,CcmC,CcmFc,CcmFn, MatR, MttB | tRNA-Met, tRNA-Tyr, tRNA-Arg, tRNA-Val, tRNA-Glu, tRNA-Gln, tRNA-Cys, tRNA-His,tRNA-Lys, tRNA-Leu， tRNA-Arg, tRNA-Val, | Rrn26, Rrn18, Rrn5 |
| 3 | Asclepias syriaca | Atp1,Atp4,Atp8,Atp9,Cob,Cox1,Cox2,Cox3,Nad1,Nad2,Nad3,Nad4,Nad4L,Nad5,Nad6,Nad7,Nad9,Rps1,Rps3,Rps4,Rps7,Rps10,Rps12,Rps13,Rps19,Rpl2,Rpl5,Rpl10,CcmB,CcmC,CcmFc,CcmFn,MatR,MttB,Sdh3,Sdh4 | tRNA-Tyr, tRNA-Lys, tRNA-Leu, tRNA-Ser, tRNA-Asp,tRNA-Gly, tRNA-Gln,tRNA-Met, tRNA-Ile, tRNA-Asn, tRNA-His, tRNA-Glu, tRNA-Phe, tRNA-Pro, tRNA-Trp, tRNA-Cys, tRNA-Thr, | Rrn26, Rrn18, Rrn5 |
| 4 | Beta macrocarpa | Atp1, Atp4,Atp6, Atp8,Atp9, Cox1,Cox2, Cox3,Nad1,Nad2, Nad3, Nad4, Nad4L,Nad5, Nad6,Nad7, Nad9, Rps3, Rps4,Rps7, Rps13, Rps12, Rpl5, Cob, CcmB, CcmFc, CcmFn, MatR,Tatc | tRNA-Ile, tRNA-Met, tRNA-Ser, tRNA-Lys, tRNA-Cys, tRNA-Pro, tRNA-Trp, tRNA-Val, tRNA-Asp, tRNA-Gln, tRNA-Gly, tRNA-Pro, tRNA-Phe, tRNA-Ser, tRNA-His, tRNA-Glu, tRNA-Asn, tRNA-Tyr, | Rrn26, Rrn18, Rrn5 |
| 5 | Beta vulgaris | Atp1,Atp6, Atp8, Atp9,Nad3, Nad4, Nad4L,Nad7, Nad9, Rps7, Cox1,Cox2, Cox3,Nad1,Nad2, Nad5, Nad6,Cob,CcmB, CcmFc, CcmFn, Rps3,Rps4, Rps12, Rps13,Rpl5, MatR,Tatc | tRNA-Met, tRNA-Ser, tRNA-Lys, tRNA-Ile, tRNA-Cys, tRNA-Pro, tRNA-Trp, tRNA-Val, tRNA-Asp, tRNA-Gln, tRNA-Gly, tRNA-Pro, tRNA-Phe, tRNA-His, tRNA-Glu, tRNA-Asn, tRNA- Tyr | Rrn26, Rrn18, Rrn5 |
| 6 | Butomus umbellatus | Atp1, Atp4,Atp6, Atp8,Atp9, Cob,Cox1, Cox2, Cox3,CcmB,CcmC, CcmFc, CcmFn, MatR, MttB, Nad1,Nad2, Nad3, Nad4, Nad4L,Nad5, Nad6, Nad7,Nad9, Rps1,Rps3, Rps7, Rps12 | tRNA-Asp, tRNA-Met, tRNA- Tyr, tRNA-Glu, tRNA-His, tRNA-Cys, tRNA-Gln, tRNA-His, tRNA-Ile, tRNA-Ugc, tRNA-Ala, tRNA-Lys, tRNA-Trp, | Rrn26,  Rrn18,  Rrn16,  Rrn5 |
| 7 | Capsicum annuum | Atp1, Atp6,Atp9, Cob,Cox1, Cox2,Cox3,CcmB,CcmC,CcmFc,CcmFn, MatR, Nad1, Nad2, Nad3,Nad4, Nad4L, Nad5, Nad6,Nad7, Nad9, Rpl2,Rpl5, Rpl16, Rpl10, Rps4, Rps10, Rps12,Rps13, Rps19,Sdh3, Sdh4 | tRNA-Met, tRNA-Leu, tRNA-Cys, tRNA-Arg, tRNA-Glu, tRNA-Gln, tRNA-Gly, tRNA-Pro, tRNA-Phe, tRNA-Ser, tRNA-Lys, tRNA-His, tRNA-Pro, tRNA-Trp, tRNA-Asn, tRNA- Tyr, tRNA-Asp, tRNA-Val | Rrn26, Rrn18, Rrn5 |
| 8 | Carica papaya | Atp1, Atp4, Atp6, Atp8, Atp9, Cob,Cox1, Cox2, Cox3, CcmB,CcmC,CcmFc, CcmFn,MatR,MttB,Nad1,Nad2, Nad3, Nad4, Nad4L,Nad5, Nad6, Nad7, Nad9, Rpl2, Rpl5, Rpl16, Rps1, Rps3,Rps4, Rps7, Rps10, Rps12,Rps13, Rps14,Rps19, Sdh3, Sdh4 | tRNA-Met,tRNA-Ser, tRNA-Phe, tRNA-Pro, tRNA-Lys,tRNA-Glu,tRNA-Gly, tRNA-Gln, tRNA- Tyr, tRNA-Asn, tRNA-Cys, tRNA-Ile, tRNA-Asp, tRNA-His, tRNA-Trp | Rrn26, Rrn18, Rrn5 |
| 9 | Citrullus lanatus | Atp1, Atp4,Atp6, Atp8, Atp9, Cob,Cox1, Cox2,Cox3, CcmB,CcmC,CcmFc,CcmFn,MatR, MttB,Nad1,Nad2, Nad3, Nad4, Nad4L, Nad5, Nad6, Nad7,Nad9,Rpl2, Rpl5,Rpl16,Rps1,Rps4,Rps7, Rps10, Rps12, Rps13,Rps19, Sdh3, Sdh4 | tRNA-Met,tRNA-Gln, tRNA-Glu,tRNA-Gly, tRNA- Tyr, tRNA-Asn, tRNA-Cys, tRNA-His, tRNA-Ile, tRNA-Lys, tRNA-Pro, tRNA-Phe, tRNA-Ser, tRNA-Thr, tRNA-Lys, tRNA-Gln, tRNA-Ser | Rrn26, Rrn18, Rrn5 |
| 10 | Cocos nucifera | Atp1,Atp4, Atp6,Atp8, Atp9, Cob, Cox1,Cox2, Cox3, CcmB,CcmC, CcmFc ，CcmFn, LhbA, MatR,MttB,Nad1, Nad2, Nad3, Nad4, Nad4L,Nad5, Nad6,Nad7,Nad9, PetB, PetG, PetL,PsbA, PsaJ,Rpo,Rpl2, Rpl5，Rpl14,Rpl16, Rpl33,Rps1,Rps2, Rps4,Rps7, Rps10, Rps11, Rps12, Rps13, Rps14,Rps19, Sdh4 | tRNA-Ile, tRNA-Asn, tRNA-Asp, tRNA-Met, tRNA-Lys, tRNA- Tyr, tRNA-Phe, tRNA-Arg, tRNA-Cys, tRNA-Gly, tRNA-Ser，tRNA-Pro, tRNA-Trp, tRNA-His | Rrn26, Rrn18, Rrn5 |
| 11 | Cucurbita pepo | Atp1, Atp4,Atp6, Atp8,Cob,Cox1, Cox2, Cox3,CcmB,CcmC, CcmFc ，CcmFn, MatR, MttB,Nad1,Nad2, Nad3,Nad4, Nad4L,Nad5, Nad6, Nad7, Nad9,Rpl2,Rpl5，Rpl16, Rps1,Rps3, Rps4,Rps7, Rps10,Rps12,Rps13, Rps19, Sdh3, Sdh4 | tRNA-Met, tRNA-Gly, tRNA-Glu, tRNA-His, tRNA-Gln, tRNA-Phe, tRNA-Pro, tRNA-Lys, tRNA-Ile, tRNA- Tyr, tRNA-Asn, tRNA-Cys, | Rrn26, Rrn18, Rrn5 |
| 12 | Cycas taitungensis | Atp1, Atp4,Atp6, Atp8,Atp9, Cob, Cox1, Cox2,Cox3, CcmB, CcmC, CcmFc ,CcmFn, MatR, MttB,Nad1,Nad2, Nad3, Nad4,Nad4L,Nad5, Nad6,Nad7, Nad9, Rpl2,Rpl5, Rps1, Rps2, Rps3, Rps4, Rps7,Rps10, Rps11,Rps12, Rps13,Rps14, Rpl16,Rps19, Sdh3 | tRNA-Leu, tRNA-Phe, tRNA-Lys, tRNA-Glu,tRNA-Gly, tRNA-Gln,tRNA-Ser，tRNA-Met, tRNA-Trp, tRNA-Asn, tRNA- Tyr, tRNA-His, tRNA-Pro, tRNA-Arg, tRNA-Ile, tRNA-Cys, tRNA-Asp, tRNA-Val | Rrn26, Rrn18, Rrn5 |
| 13 | Daucus carota | Atp1, Atp4,Atp6, Atp8,Atp9, CcmB, CcmC,CcmFc, Cob,Cox1,Cox2,Cox3, CcmFn,MatR, MttB,Nad1, Nad2, Nad3,Nad4, Nad4L,Nad5, Nad6, Nad7,Nad9, Rpl2, Rpl5, Rpl0,Rpl16,Rps1, Rps3, Rps4, Rps7,Rps12, Rps13 | tRNA-Trp, tRNA-Gln, tRNA-Gly, tRNA-Asp, tRNA-Met, tRNA-His, tRNA-Glu, tRNA-Ser，tRNA-Pro, tRNA-Phe, tRNA-Lys, tRNA-Asn, tRNA- Tyr, tRNA-Pro, tRNA-Phe | Rrn26, Rrn18, Rrn5 |
| 14 | Geranium maderense | Atp1,Atp4, Atp6, Atp8,Atp9,Cob,Cox1,Cox2,Cox3,CcmB,CcmC,CcmFc, CcmFn, MatR,Nad1, Nad2, Nad3,Nad4, Nad4L,Nad5, Nad6,Nad7, Nad9, Rpl10, Rps1,Rps3,TatC， | tRNA-Met, tRNA-Thr, tRNA-Val, tRNA-Ser，tRNA-Phe, tRNA-Pro, tRNA-Glu, tRNA-Asn, tRNA-His, tRNA-Cys, tRNA-Asn, tRNA-Trp, tRNA-Leu, tRNA-Gly, tRNA-Trp, tRNA-Asp, tRNA-Ile, tRNA-Gln, tRNA-Lys, tRNA- Tyr, | Rrn5  RrnL,  RrnS |
| 15 | Ginkgo biloba | Atp1,Atp4, Atp6, Atp8,Atp9, Cob,Cox1,Cox2, Cox3,CcmB, CcmC, CcmFc,CcmFn, MatR, MttB,Nad1, Nad2, Nad3, Nad4, Nad4L,Nad5, Nad6,Nad7,Nad9,Rpl2, Rpl5,Rpl16, Rps1,Rps2, Rps3, Rps4, Rps7, Rps10, Rps11, Rps12,Rps13, Rps14, Rps19, Sdh3, Sdh4 | tRNA-Trp, tRNA-Asp, tRNA- Tyr, tRNA-Phe, tRNA-Ser，tRNA-Leu, tRNA-Pro, tRNA-Cys, tRNA-Ser，tRNA-Lys, tRNA-Met, tRNA-Gln, tRNA-Arg, tRNA-His | Rrn26, Rrn18, Rrn5 |
| 16 | Glycine max | Atp1,Atp4, Atp6, Atp8,Atp9, Cob, Cox1, Cox2, Cox3, CcmB,CcmC, CcmFc ，CcmFn, MatR, MttB,Nad1, Nad2, Nad3, Nad4, Nad4L, Nad5,Nad6, Nad7,Rpl5, Rpl16,Rps1, Rps3, Rps4, Rps10,Rps12,Rps14 | tRNA-Trp, tRNA-Met, tRNA-Glu, tRNA-Ile, tRNA-Ser，tRNA-Phe，tRNA-Pro, tRNA-Asn, tRNA- Tyr, tRNA-Lys, tRNA-Gln, tRNA-Gly, tRNA-Asp, tRNA-His, tRNA-Cys | Rrn5,  RrnL,  RrnS |
| 17 | Hyoscyamus niger | Atp1, Atp4,Atp6, Atp8,Atp9, Cob, Cox1, Cox2, Cox3, CcmB, CcmC,CcmFc ，CcmFn, MatR, MttB, Nad1, Nad2, Nad3, Nad4, Nad4L,Nad5, Nad6,Nad7, Nad9, Rpl2, Rpl5, Rpl0,Rpl16, Rps1,Rps3, Rps4,Rps10, Rps12, Rps13, Rps19, Sdh3, Sdh4 | tRNA-Leu, tRNA-Ile, tRNA-Pro, tRNA-Phe，tRNA-Ser, tRNA-Met, tRNA-Gln, tRNA-Trp, tRNA-His, tRNA-Gly, tRNA-Lys, tRNA- Tyr, tRNA-Asn, tRNA-Cys, tRNA-Asp, tRNA-Glu | Rrn5,  RrnL,  RrnS |
| 18 | Ipomoea nil | Atp1, Atp4, Atp6, Atp8,CcmB,CcmC, CcmFn, Cox1, Cox2, Cox3, MatR, Nad1, Nad2, Nad3, Nad4, Nad5, Nad6, Nad7, Nad9, Rpl5,Rpl10,Rpl16, Rps1, Rps3, Rps4, Rps12, Rps13, Rps14, Rps19, Sdh4 | tRNA- Tyr, tRNA-Asn, tRNA-Gly, tRNA-Cys, tRNA-Met, tRNA-His, tRNA-Ile, tRNA-Pro, tRNA-Phe，tRNA-Ser, tRNA-Lys, tRNA-Asp, tRNA-Glu, tRNA-Gln, tRNA-Trp | Rrn5,  RrnL,  RrnS |
| 19 | Liriodendron tulipifera | Atp1,Atp4,Atp6,Atp8, Atp9, CcmB,CcmC,CcmFc,CcmFn, Cob, Cox1,Cox2, Cox3, MatR, MttB, Nad1, Nad2, Nad3, Nad4, Nad4L,Nad5,Nad6, Nad7, Nad9, Rpl2,Rpl5, Rpl10,Rpl16, Rps1,Rps2, Rps3, Rps4, Rps7, Rps10,Rps11, Rps12, Rps13,Rps14, Rps19, Sdh3,Sdh4 | tRNA-Asn, tRNA-Met, tRNA-Gln, tRNA-Asp, tRNA-Ile, tRNA-Ser, tRNA-Phe，tRNA-Pro, tRNA-Gly, tRNA-Glu, tRNA-Ser, tRNA-Trp, tRNA-Val,tRNA-Lys, tRNA-Cys, tRNA-His, tRNA- Tyr | Rrn5,  RrnL,  RrnS |
| 20 | Malus domestica | Atp1,Atp6,Atp8,Atp9,CcmB,CcmC,CcmFc, CcmFn, Cob,Cox1, Cox2,Cox3, MatR,Nad1, Nad2,Nad3,Nad4, Nad4L, Nad5, Nad6, Nad7, Nad9,RnaseH,Rpl5, Rps1, Rps3,Rps12, Rps13,Rps14, Sdh4 | tRNA-Ile, tRNA-Asp, tRNA-Phe，tRNA-Lys, tRNA-Glu, tRNA-His, tRNA-Met, tRNA-Gly, tRNA-Gln, tRNA- Tyr, tRNA-Asn, tRNA-Cys, tRNA-Pro, tRNA-Trp, tRNA-Ser | Rrn26, Rrn18, Rrn5 |
| 21 | Medicago truncatula | Atp1, Atp4, Atp6, Atp8, Atp9,CcmB, CcmC, CcmFc,CcmFn, Cob, Cox1, Cox3,MatR, MttB, Nad1, Nad2, Nad3,Nad4, Nad4L,Nad5, Nad6, Nad7,Nad9, Rpl5,Rpl16,Rps1, Rps3, Rps4,Rps10, Rps12, Rps14 | tRNA-Met, tRNA-Phe，tRNA-Pro, tRNA-Lys, tRNA-Glu, tRNA-Ile, tRNA-Gly, tRNA-Gln, tRNA-Trp, tRNA-His, tRNA-Asp, tRNA-Cys, tRNA-Asn, tRNA-Tyr | Rrn5,  RrnL,  RrnS |
| 22 | Millettia pinnata | Atp1, Atp4, Atp6, Atp8, Atp9,CcmB, CcmC, CcmFc,CcmFn, Cob, Cox1, Cox2,Cox3,MatR, MttB, Nad1, Nad2, Nad3,Nad4, Nad4L,Nad5, Nad6, Nad7,Nad9, Rpl5,Rpl16,Rps1, Rps3, Rps4,Rps10, Rps12, Rps14 Sdh3 | tRNA-Met, tRNA-Phe，tRNA-Pro, tRNA-Lys, tRNA-Glu, tRNA-Ile, tRNA-Gly, tRNA-Gln, tRNA-Trp, tRNA-His, tRNA-Asp, tRNA-Cys, tRNA-Asn, tRNA-Tyr,  tRNA-Thr, tRNA-Lys  tRNA-Ser | Rrn26, Rrn18, Rrn5 |
| 23 | Nelumbo nucifera | Atp1,Atp4, Atp6, Atp8, Atp9, CcmB,CcmC, CcmFc,CcmFn,Cob,Cox1,Cox2, Cox3,MatR,Nad1, Nad2, Nad3, Nad4,Nad4L , Nad5,Nad6, Nad7, Nad9, PetN,Rpl2, Rpl5, Rpl10, Rpl16,Rps1,Rps2, Rps3,Rps4, Rps7, Rps10, Rps11, Rps12, Rps13, Rps14, Rps19,Sdh3, Sdh4 | tRNA-Met, tRNA-Glu, tRNA-Gly, tRNA-Cys, tRNA-Asn, tRNA-Tyr, tRNA-Lys, tRNA-Gln, tRNA-Asp, tRNA-Thr, tRNA-Ser, tRNA-His, tRNA-Phe, tRNA-Pro, tRNA-Trp | Rrn26, Rrn18, Rrn5 |
| 24 | Phoenix dactylifera | Atp1,Atp4, Atp6, Atp8, Atp9, Cob, Cox1, Cox2,Cox3, CcmB, CcmC,CcmFc, CcmFn, MatR,MttB,Nad1,Nad2, Nad3,Nad4,Nad4L ，Nad5, Nad6,Nad7, Nad9,Rpl2,Rpl5, Rpl16, Rps1, Rps2, Rps3, Rps4, Rps7, Rps11, Rps12,Rps13,Rps14, Rps19, | tRNA-Gln, tRNA-Met, tRNA-Glu, tRNA-Asp, tRNA-Lys, tRNA-Cys, tRNA-Pro, tRNA-Ser, tRNA-Thr, tRNA-Tyr, | Rrn26, Rrn18, Rrn5 |
| 25 | Raphanus sativus | Atp1,Atp4,Atp6, Atp8,Atp9, CcmB,CcmC,CcmFc,CcmFn, Cob, Cox1,Cox2, Cox3,MatR,Nad1, Nad2, Nad3, Nad4,Nad4L ，Nad5, Nad6, Nad7, Nad9, Rpl2,Rpl5, Rpl16,Rps3, Rps4,Rps7, Rps12,Rps14, | tRNA-Cys, tRNA-Pro, tRNA-Glu, tRNA-Trp, tRNA-Ser, tRNA-His, tRNA-Gln, tRNA-Met, tRNA-Ile, tRNA-Asp, tRNA-Lys, tRNA-Gly, tRNA-Tyr, tRNA-Asn | Rrn26, Rrn18, Rrn5 |
| 26 | Salix purpurea | Atp1,Atp4,Atp6, Atp8, Atp9, CcmB,CcmC, CcmFc, CcmFn, Cob,Cox1, Cox2, Cox3, MatR,MttB,Nad1, Nad2, Nad3, Nad4, Nad4L ，Nad5, Nad6,Nad7, Nad9, Rpl2,Rpl16,Rpll0,Rps4,Rps7,Rps12, Rps3, Sdh4 | tRNA-Val, tRNA-Met, tRNA-Ser, tRNA-Asp, tRNA-Lys, tRNA-Pro, tRNA-His, tRNA-Cys, tRNA-Asn, tRNA-Tyr, tRNA-Phe, tRNA-Trp, tRNA-Glu, tRNA-Gln, tRNA-Gly | Rrn5,  RrnL,  RrnS |
| 27 | Salix suchowensis | Atp1, Atp4, Atp6,Atp8, Atp9, CcmB,CcmC,CcmFc,CcmFn, Cob,Cox1, Cox2,Cox3, MatR, MttB,Nad1,Nad2, Nad3, Nad4, Nad4L ，Nad5, Nad6,Nad7, Nad9,Rpl2,Rpll0, Rpl16, Rps3, Rps4,Rps7, Rps12,Sdh4 | tRNA-Met, tRNA-Val, tRNA-Lys, tRNA-Tyr, tRNA-Asn, tRNA-Cys, tRNA-His, tRNA-Phe, tRNA-Ser, tRNA-Trp, tRNA-Glu, tRNA-Gln, tRNA-Gly, tRNA-Asp | Rrn5,  RrnL,  RrnS |
| 28 | Salvia miltiorrhiza | Atp1, Atp4, Atp6, Atp8,Atp9, AtpB, AtpE, CcmB,CcmC, CcmFc, CcmFn, Cob, Cox1,Cox2, Cox3, MatR, MttB,Nad1, Nad2, Nad3,Nad4,Nad4L ，Nad5,Nad6, Nad7, Nad9,PetG,PetL,RbcL, Rpl2, Rpl5, Rpll0, Rpl16, Rpl23, Rps3, Rps4,Rps7,Rps10, Rps12,Rps13,Rps14, | tRNA-Met, tRNA-Trp, tRNA-Cys, tRNA-Asn, tRNA-Tyr, tRNA-Pro, tRNA-Phe, tRNA-Ser, tRNA-Glu, tRNA-Ile, tRNA-Leu, tRNA-Val, tRNA-His, tRNA-Gln, tRNA-Asp, tRNA-Gly, tRNA-Lys | Rrn26, Rrn18, Rrn5 |
| 29 | Sorghum bicolor | Atp1, Atp4, Atp6,Atp8, Atp9, CcmFc, CcmFn, CcmB,CcmC,Cob, Cox1, Cox2,Cox3, MatR, MttB,Nad1, Nad2, Nad3, Nad4,Nad4L ， Nad5, Nad6, Nad7,Nad9, Rpl16, Rps1, Rps2, Rps3, Rps4, Rps7,Rps12, Rps13 | tRNA-Tyr, tRNA-Phe, tRNA-Ser, tRNA-Ile, tRNA-Asp, tRNA-Asn, tRNA-Lys, tRNA-Met, tRNA-Cys, tRNA-Gln, tRNA-Glu, tRNA-Pro, tRNA-Trp, tRNA-His | Rrn26, Rrn18, Rrn5 |
| 30 | Triticum aestivum | Atp1, Atp4,Atp6, Atp8, Atp9,CcmB,CcmFc, CcmFn,Cob, Cox1,Cox2, Cox3, MatR, MttB,Nad1,Nad2,Nad3,Nad4, Nad4L ，Nad5, Nad6, Nad7,Nad9, Rpl5, Rpl16, Rps1, Rps2, Rps3, Rps4, Rps7,Rps12, Rps13,Rps19 | tRNA-Glu, tRNA-Met, tRNA-Pro, tRNA-Gln, tRNA-Lys, tRNA-Tyr, tRNA-Asp, tRNA-Ser, tRNA-Phe, tRNA-Asn, tRNA-Trp, tRNA-His | Rrn26, Rrn18, Rrn5 |
| 31 | Vaccinium macrocarpon | Atp1, Atp4, Atp8, Atp9, CcmB, CcmC, CcmFc, CcmFn, Cob,Cox1, Cox2, Cox3, MatR,MttB, Nad1,Nad2, Nad3, Nad4, Nad5, Nad6, Nad7,Nad9, Rpl2, Rpl10,Rpl16, Rps1,Rps4, Rps10, Rps12, Rps13, Rps15,Rps19, Sdh4 | tRNA-Met, tRNA-Trp,  tRNA-Lys, tRNA-Pro, tRNA-Phe, tRNA-Ser, tRNA-Cys, tRNA-Asn, tRNA-Tyr, tRNA-Leu, tRNA-Ile, tRNA-Asp, tRNA-Gln, tRNA-His, tRNA-Glu | Rrn26, Rrn18, Rrn5 |
| 32 | Vigna angularis | Atp1, Atp4, Atp6,Atp8, Atp9, CcmB, CcmC, CcmFn, Cob,Cox1,Cox3, MatR,Nad2, Nad3, Nad4,Nad6, Nad7, Nad9, Rpl5,Rpl16,Rps1, Rps3, Rps4, Rps12, Rps14 | tRNA-Phe, tRNA-Pro, tRNA-Met, tRNA-Glu, tRNA-Ile, tRNA-Trp, tRNA-Tyr, tRNA-Asn, tRNA-Met, tRNA-Gly, tRNA-Gln, tRNA-Lys, tRNA-Cys, tRNA-His | Rrn5,  RrnL,  RrnS |
| 33 | Vigna radiata | Atp1, Atp4, Atp6, Atp8,Atp9, CcmB, CcmC, CcmFc, CcmFn,Cob,Cox1, Cox3, MatR, MttB, Nad1, Nad2, Nad3, Nad4, Nad4L ，Nad5, Nad6, Nad7,Nad9,Rpl5,Rpl16, Rps1,Rps3, Rps4, Rps10, Rps12, Rps14 | tRNA-Met, tRNA-Glu, tRNA-Ile, tRNA-Trp, tRNA-Asn, tRNA-Tyr, tRNA-Phe, tRNA-Pro, tRNA-Lys, tRNA-Cys, tRNA-His,tRNA-Gln, tRNA-Gly, tRNA-Cys, | Rrn5,  RrnL,  RrnS |
| 34 | Vitis vinifera | Atp1,Atp4, Atp6,Atp8, Atp9, AtpA,CcmB, CcmC, CcmFc,CcmFn, Cob, Cox1,Cox2,Cox3,InfA, MatR,Nad1, Nad2,Nad3, Nad4,Nad4L, Nad5,Nad6, Nad7,Nad9, NdhE,PetA,PsaC, PsaJ, PsbM,PetL,PetG PsbA, PsbJ, PsbN,PsbT,RbcL,Rpl2, Rpl5,Rpl14, Rpl16, Rpl20, Rpl32, Rpl33, Rpl36,Rps1, Rps3, Rps4, Rps7,Rps10, Rps11,Rps12, Rps13, Rps14,Rps15, Rps18, Rps19, Sdh3,Sdh4,Ycf4 | tRNA-Gln, tRNA-Gly, tRNA-Asp, tRNA-Asn, tRNA-Glu, tRNA-His, tRNA-Lys, tRNA-Ser, tRNA-Phe, tRNA-Pro, tRNA-Trp, tRNA-Ile, tRNA-Leu, tRNA-Cys, tRNA-Tyr, tRNA-Arg, tRNA-Thr | Rrn26, Rrn18, Rrn5 |
| 35 | Welwitschia mirabilis | Atp1, Atp4, Atp6, Atp8, Atp9,CcmB, CcmC, CcmFc, CcmFn, Cob,Cox1,Cox2, Cox3, MatR,MttB, Nad1,Nad2, Nad3, Nad4, Nad5,Nad4L, Nad6, Nad7, Nad9, Rpl10, Rps3,Rps4, Rps12, Sdh4 | tRNA-Glu, tRNA-Asp, tRNA-Trp, tRNA-Ile, tRNA-Gln, tRNA-Met, tRNA-Arg, tRNA-Tyr | Rrn26, Rrn18, Rrn5 |
| 36 | Zea luxurians | Atp1, Atp4,Atp6, Atp8, Atp9,CcmB,CcmC, CcmFc, CcmFn, Cob, Cox1, Cox2,Cox3, MatR, MttB, Nad1, Nad2, Nad3, Nad4,Nad4L, Nad5, Nad6, Nad7, Nad9,Rpl6, Rpl16, Rps1,Rps2,Rps3,Rps4,Rps7,Rps12, Rps13 | tRNA-Asn, tRNA-Asp, tRNA-Ile, tRNA-Glu, tRNA-Pro, tRNA-Met, tRNA-Tyr, tRNA-Lys, tRNA-His, tRNA-Gln, tRNA-Ser, tRNA-Cys, tRNA-Phe | Rrn26, Rrn18, Rrn5 |
| 37 | Zea perennis | Atp1,Atp4, Atp6, Atp8,Atp9, CcmB,CcmC, CcmFc, CcmFn,Cob, Cox1,Cox2, Cox3, MatR,MttB, Nad1,Nad2,Nad3, Nad4, Nad4L, Nad5, Nad6, Nad7, Nad9,Rpl16, Rps1,Rps2,Rps3, Rps4, Rps7,Rps12, Rps13 | tRNA-Met, tRNA-Ser, tRNA-Gln, tRNA-Pro, tRNA-His, tRNA-Cys, tRNA-Phe, tRNA-Asn, tRNA-Asp, tRNA-Ile, tRNA-Glu, tRNA-Pro, tRNA-Tyr, tRNA-Lys | Rrn26, Rrn18, Rrn5 |
| 38 | Ziziphus jujuba | Atp1,Atp4, Atp6, Atp8,Atp9, CcmB,CcmC, CcmFc, CcmFn, Cox1,Cox2, Cox3, MatR,MttB, Nad1,Nad2,Nad3, Nad4, Nad4L, Nad5, Nad6, Nad7, Nad9, Rpl5, Rpl10, Rpl16, Rps1,Rps3, Rps7, Rps10,Rps12, Rps13，Rps19，Sdh3,Sdh4 | tRNA-Met, tRNA-Gly, tRNA-Ser, tRNA-Gln, tRNA-Pro, tRNA-His, tRNA-Cys, tRNA-Phe, tRNA-Asn, tRNA-Asp, tRNA-Ile, tRNA-Glu, tRNA-Tyr, tRNA-Lys, tRNA-Val,tRNA-Trp, tRNA-Cys, | Rrn26,  Rrn5 |
| 39 | Zea mays | Atp1,Atp4, Atp6, Atp8,Atp9, CcmB,CcmC, CcmFc, CcmFn, Cob,Cox1,Cox2, Cox3, MatR,MttB, Nad1,Nad2,Nad3, Nad4, Nad4L, Nad5, Nad6, Nad7, Nad9, Rpl16, Rps1, Rps2,Rps3, Rps4, Rps7, Rps12, Rps13， | tRNA-Met, tRNA-Ser, tRNA-Gln, tRNA-Pro, tRNA-His, tRNA-Cys, tRNA-Phe, tRNA-Asn, tRNA-Asp, tRNA-Ile, tRNA-Glu, tRNA-Tyr, tRNA-Lys, tRNA-Cys | Rrn26, Rrn18, Rrn5 |
