# Supplementary material for: Organellar genome assembly methods and comparative analysis of horticultural plants
Source: Hortic Res. 2018 Jan 10;5:3. doi: 10.1038/s41438-017-0002-1 (PMC5798811; doi:10.1038/s41438-017-0002-1)
Supplement: Supplementary file 2 — Table S2 [file 41438_2017_2_MOESM2_ESM.docx]

Table S2 Overview of the 39 horticultural mitochondrial genome sequences

|  | Species | Length(bp) | A(bp) | T(bp) | A+T content(%) |
| --- | --- | --- | --- | --- | --- |
| 1 | Ajuga reptans | 352,069 | 96,253 | 97,043 | 54.90 |
| 2 | Allium cepa | 316,363 | 86,944 | 85,957 | 54.65 |
| 3 | Asclepias syriaca | 682,498 | 193755 | 192356 | 56.57 |
| 4 | Beta macrocarpa | 385,220 | 108,829 | 107,307 | 56.11 |
| 5 | Beta vulgaris | 364,950 | 103,006 | 101,704 | 56.09 |
| 6 | Butomus umbellatus | 450,826 | 115,382 | 114,088 | 50.90 |
| 7 | Capsicum annuum | 511,530 | 142,872 | 140,901 | 55.48 |
| 8 | Carica papaya | 476,890 | 131,172 | 130,559 | 54.88 |
| 9 | Citrullus lanatus | 379,236 | 103,995 | 104,286 | 54.92 |
| 10 | Cocos nucifera | 678,653 | 183,712 | 186,002 | 54.48 |
| 11 | Cucurbita pepo | 982,833 | 280,581 | 281,635 | 57.20 |
| 12 | Cycas taitungensis | 414,903 | 109,699 | 110,536 | 53.08 |
| 13 | Daucus carota | 281,132 | 76,481 | 76,965 | 54.58 |
| 14 | Geranium maderense | 737,091 | 210,897 | 214,286 | 57.68 |
| 15 | Ginkgo biloba | 346,544 | 84,773 | 87,258 | 49.64 |
| 16 | Glycine max | 402,558 | 110,241 | 111,042 | 54.97 |
| 17 | Hyoscyamus niger | 501,401 | 137,230 | 137,639 | 54.82 |
| 18 | Ipomoea nil | 265,768 | 74,370 | 73,265 | 55.55 |
| 19 | Liriodendron tulipifera | 553,721 | 144,376 | 145,199 | 52.30 |
| 20 | Malus domestica | 396,947 | 108,379 | 108,303 | 54.59 |
| 21 | Medicago truncatula | 271,618 | 73,899 | 74,433 | 54.61 |
| 22 | Millettia pinnata | 425,718 | 116,747 | 117,385 | 55.00 |
| 23 | Nelumbo nucifera | 524,797 | 136,766 | 135,313 | 51.84 |
| 24 | Phoenix dactylifera | 715,001 | 198,081 | 194,139 | 54.86 |
| 25 | Raphanus sativus | 258,426 | 71,113 | 70,469 | 54.79 |
| 26 | Salix purpurea | 598,970 | 163,167 | 166,640 | 55.06 |
| 27 | Salix suchowensis | 644,437 | 176,766 | 177,780 | 55.02 |
| 28 | Salvia miltiorrhiza | 499,236 | 139,131 | 138,506 | 55.61 |
| 29 | Sorghum bicolor | 468,628 | 131,480 | 132,231 | 56.27 |
| 30 | Triticum aestivum | 452,528 | 126,114 | 125,709 | 55.65 |
| 31 | Vaccinium macrocarpon | 459,678 | 125,718 | 125,567 | 54.67 |
| 32 | Vigna angularis | 404,466 | 110,992 | 110,705 | 54.81 |
| 33 | Vigna radiate | 401,262 | 110,248 | 109,999 | 54.89 |
| 34 | Vitis vinifera | 773,279 | 216,224 | 215,694 | 55.86 |
| 35 | Welwitschia mirabilis | 978,846 | 230,428 | 229,424 | 46.98 |
| 36 | Zea luxurians | 539,368 | 151,388 | 151,016 | 56.07 |
| 37 | Zea perennis | 570,354 | 159,936 | 160,119 | 56.12 |
| 38 | Ziziphus jujuba | 365,190 | 99,774 | 100,079 | 54.73 |
| 39 | Zea mays | 680,603 | 190,935 | 191,025 | 56.12 |
