# Supplementary material for: Organellar genome assembly methods and comparative analysis of horticultural plants
Source: Hortic Res. 2018 Jan 10;5:3. doi: 10.1038/s41438-017-0002-1 (PMC5798811; doi:10.1038/s41438-017-0002-1)
Supplement: Supplementary file 3 — Table S3 [file 41438_2017_2_MOESM3_ESM.docx]

Table S3 Overview of the 34 horticultural chloroplast genome sequences

|  | Species | Length(bp) | A(bp) | T(bp) | A+T content (%) |
| --- | --- | --- | --- | --- | --- |
| 1 | Ajuga reptans | 149,963 | 45,779 | 46,768 | 61.71 |
| 2 | Allium cepa | 153,538 | 48,048 | 48,975 | 63.19 |
| 3 | Asclepias syriaca | 158,719 | 48,981 | 49,696 | 62.17 |
| 4 | Beta vulgaris | 149,635 | 46,711 | 47,559 | 63.03 |
| 5 | Capsicum annuum | 156,781 | 48,212 | 49,423 | 62.27 |
| 6 | Carica papaya | 160,100 | 49,931 | 51,107 | 63.11 |
| 7 | Citrullus lanatus | 156,906 | 48,563 | 50,007 | 62.82 |
| 8 | Cocos nucifera | 154,731 | 47,937 | 48,864 | 62.56 |
| 9 | Cycas taitungensis | 163,403 | 48,756 | 50,173 | 60.54 |
| 10 | Daucus carota | 155,911 | 48,106 | 49,096 | 62.34 |
| 11 | Geranium maderense | 155,694 | 46,875 | 48,073 | 60.98 |
| 12 | Ginkgo biloba | 156,988 | 46,773 | 48,108 | 60.44 |
| 13 | Glycine max | 152,218 | 49,270 | 49,104 | 64.63 |
| 14 | Hyoscyamus niger | 155,720 | 48,046 | 49,174 | 62.43 |
| 15 | Ipomoea nil | 161,897 | 50,340 | 50,902 | 62.53 |
| 16 | Liriodendron tulipifera | 159,886 | 47,995 | 49,284 | 60.84 |
| 17 | Medicago truncatula | 124,033 | 40,850 | 41,046 | 66.03 |
| 18 | Millettia pinnata | 152,968 | 49,798 | 49,897 | 65.17 |
| 19 | Nelumbo nucifera | 163,330 | 50,120 | 51,188 | 62.03 |
| 20 | Phoenix dactylifera | 158,462 | 49,280 | 50,182 | 62.77 |
| 21 | Raphanus sativus | 153,368 | 48,078 | 49,557 | 63.66 |
| 22 | Salix purpurea | 155,590 | 49,845 | 48,666 | 63.31 |
| 23 | Salix suchowensis | 155,214 | 49,722 | 48,476 | 63.27 |
| 24 | Salvia miltiorrhiza | 151,328 | 46,368 | 47,424 | 61.98 |
| 25 | Sorghum bicolor | 140,754 | 43,351 | 43,225 | 61.51 |
| 26 | Triticum aestivum | 134,545 | 41,666 | 41,329 | 61.69 |
| 27 | Vigna angularis | 151,683 | 49,360 | 48,952 | 64.81 |
| 28 | Vigna radiate | 151,271 | 49,195 | 48,776 | 64.77 |
| 29 | Vitis vinifera | 160,928 | 49,793 | 50,951 | 62.60 |
| 30 | Welwitschia mirabilis | 119,726 | 37,435 | 38,321 | 63.27 |
| 31 | Zea luxurians | 140,710 | 43,404 | 43,222 | 61.56 |
| 32 | Zea perennis | 140,647 | 43,388 | 43,187 | 61.55 |
| 33 | Ziziphus jujuba | 161,466 | 50,387 | 51,683 | 63.21 |
| 34 | Zea mays | 140,384 | 43,281 | 43,108 | 61.54 |

Note: The chloroplast genome of *Beta macrocarpa*, *Butomus umbellatus*, *Cucurbita pepo*, *Cucurbita pepo*, *Malus domestica* and *Vaccinium macrocarpon* have not been published in NCBI.
