# Supplementary figures and images for: Organellar genome assembly methods and comparative analysis of horticultural plants
Source: Hortic Res. 2018 Jan 10;5:3. doi: 10.1038/s41438-017-0002-1 (PMC5798811; doi:10.1038/s41438-017-0002-1)

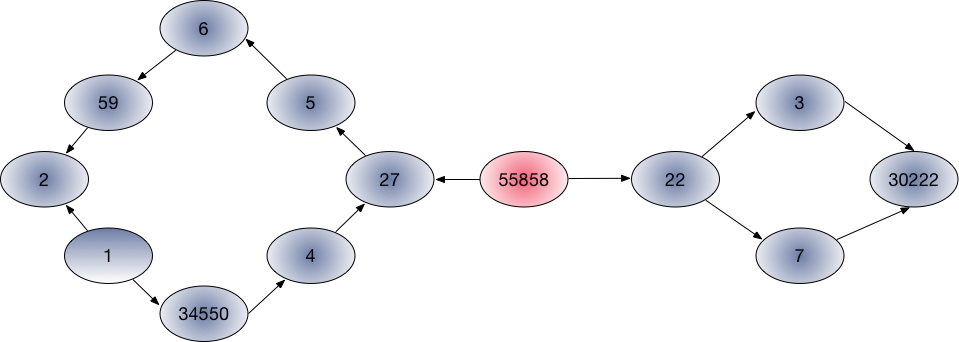

Supplement: Supplementary file 4 — Figure S1 [file 41438_2017_2_MOESM4_ESM.jpg]
